# The Role of Complement in Cnidarian-Dinoflagellate Symbiosis and Immune Challenge in the Sea Anemone *Aiptasia pallida*

**DOI:** 10.3389/fmicb.2016.00519

**Published:** 2016-04-22

**Authors:** Angela Z. Poole, Sheila A. Kitchen, Virginia M. Weis

**Affiliations:** ^1^Department of Integrative Biology, Oregon State UniversityCorvallis, OR, USA; ^2^Department of Biology, Western Oregon UniverstiyMonmouth, OR, USA

**Keywords:** *Aiptasia*, symbiosis, complement, innate immunity, cnidarians, *Symbiodinium*, *Serratia marcescens*

## Abstract

The complement system is an innate immune pathway that in vertebrates, is responsible for initial recognition and ultimately phagocytosis and destruction of microbes. Several complement molecules including C3, Factor B, and mannose binding lectin associated serine proteases (MASP) have been characterized in invertebrates and while most studies have focused on their conserved role in defense against pathogens, little is known about their role in managing beneficial microbes. The purpose of this study was to (1) characterize complement pathway genes in the symbiotic sea anemone *Aiptasia pallida*, (2) investigate the evolution of complement genes in invertebrates, and (3) examine the potential dual role of complement genes Factor B and MASP in the onset and maintenance of cnidarian-dinoflagellate symbiosis and immune challenge using qPCR based studies. The results demonstrate that *A. pallida* has multiple Factor B genes (Ap_Bf-1, Ap_Bf-2a, and Ap_Bf-2b) and one MASP gene (Ap_MASP). Phylogenetic analysis indicates that the evolutionary history of complement genes is complex, and there have been many gene duplications or gene loss events, even within members of the same phylum. Gene expression analyses revealed a potential role for complement in both onset and maintenance of cnidarian-dinoflagellate symbiosis and immune challenge. Specifically, Ap_Bf-1 and Ap_MASP are significantly upregulated in the light at the onset of symbiosis and in response to challenge with the pathogen *Serratia marcescens* suggesting that they play a role in the initial recognition of both beneficial and harmful microbes. Ap_Bf-2b in contrast, was generally downregulated during the onset and maintenance of symbiosis and in response to challenge with *S. marcescens*. Therefore, the exact role of Ap_Bf-2b in response to microbes remains unclear, but the results suggest that the presence of microbes leads to repressed expression. Together, these results indicate functional divergence between Ap_Bf-1 and Ap_Bf-2b, and that Ap_Bf-1 and Ap_MASP may be functioning together in an ancestral hybrid of the lectin and alternative complement pathways. Overall, this study provides information on the role of the complement system in a basal metazoan and its role in host-microbe interactions.

## Introduction

The immune system is classically thought of as the mechanisms that defend an organism against harmful intruders, such as disease-causing microbes or parasitic worms. Although the word microbe is traditionally associated with pathogenic organisms, a more modern interpretation is that one microbe can be harmful or beneficial depending on the host or changing environmental conditions (Eberl, 2010). In addition, it has been recognized that beneficial and harmful microbes both interact with immune pathways to persist within the host (Hentschel et al., 2000; Kubinak and Round, 2012). Therefore, it has been proposed that the immune system functions more generally to maintain homeostasis by defining the microbial community through interactions with both mutualistic and pathogenic microbes (Eberl, 2010).

Many invertebrates engage in mutualistic symbiotic relationships with bacteria and other microbes and therefore require mechanisms to allow for tolerance of mutualists and elimination of pathogens. Invertebrates, unlike vertebrates, lack the highly specific adaptive immune response and depend on innate immune pathways to mediate these interactions (McFall-Ngai, 2007). The innate immune system represents the fast-acting, relatively non-specific mechanisms that recognize and respond to non-self-molecules (Akira et al., 2006; Iwasaki and Medzhitov, 2010). Many innate immune pathways are initiated by interactions between host pattern recognition receptors (PRRs) and general microbial signatures called microbe associated molecular patterns (MAMPs) (Medzhitov and Janeway, 1997; Eberl, 2010). PRRs include extracellular receptors such as Toll-like receptors (TLRs), scavenger receptors (SRs), lectins, and complement receptors, and also the intracellular nod-like receptors (NLRs) (Janeway and Medzhitov, 2002). Ultimately, these interactions activate signaling cascades to initiate the appropriate response to a microbe.

**Figure 1:**
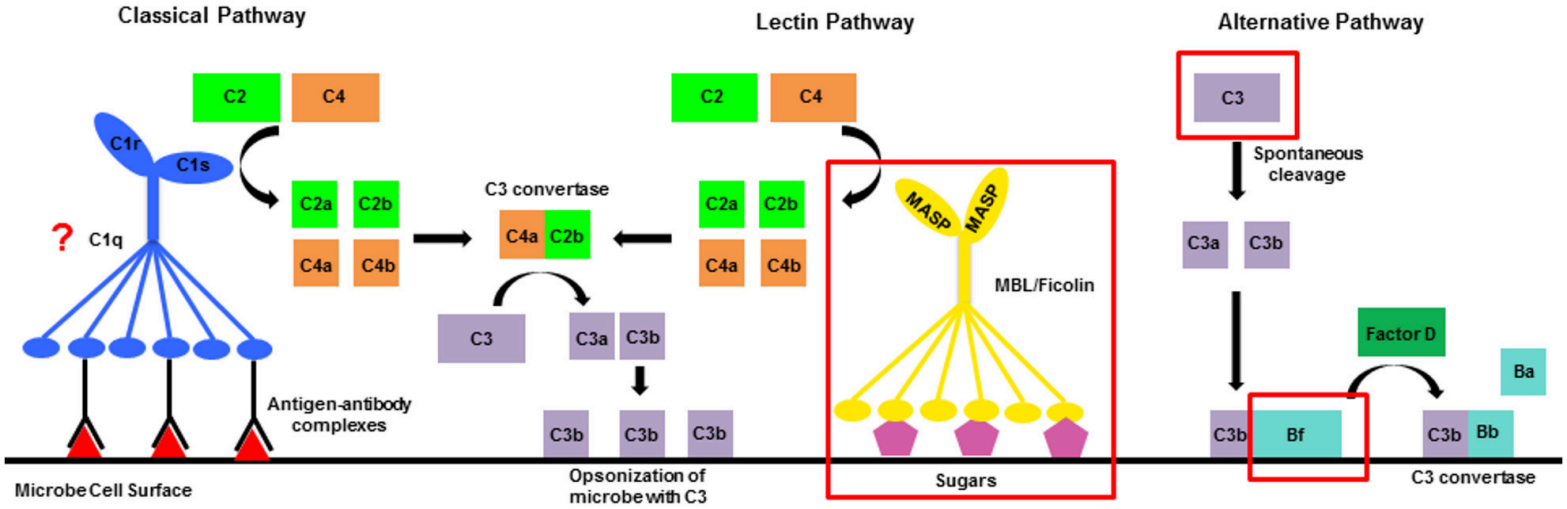
**Diagrammatic representation of the complement system**. In vertebrates there are three activation mechanisms including the classical, lectin, and alternative pathways, which all converge at cleavage of the central protein C3. Invertebrates only possess components of the lectin and alternative pathways. Red boxes surround sequences that have been characterized in invertebrate genomes and transcriptomes. The question mark by C1q indicates that proteins with the C1q domain have been characterized in invertebrates, but a direct link to their role in the complement system has not been established.

The complement system is one innate immune pathway that can be activated by the interaction of PRRs and MAMPs. It is involved in initial detection of microbes and subsequent activation of a series of proteolytic cleavage events that ultimately result in phagocytosis and destruction of microbes and other foreign cells (Gros et al., 2008). In vertebrates, this response can be activated by three distinct mechanisms: the classical, lectin, and alternative pathways. All three use different recognition molecules, but converge at cleavage of the central molecule in the pathway, C3, to C3a and C3b by a protein complex called the C3 convertase (Gros et al., 2008; Mayilyan et al., 2008; Figure 1). Downstream of C3 cleavage, C3a diffuses away to activate an inflammatory response. C3b remains attached to the surface of the microbe where it serves as an opsonin that binds complement receptors to initiate phagocytosis and activates events that lead to the formation of the membrane attack complex (MAC) and ultimately to cell lysis (Ehlenberger and Nussenzweig, 1977; Pangburn and Rawal, 2002; Gros et al., 2008).

The complement system in invertebrates is less elaborate than vertebrate pathways as it contains just a few of the key initiation molecules and lacks most proteins involved in formation of the MAC (Figure 1; Cerenius et al., 2010). This suggests that the primary function of the ancestral complement pathway may have been to promote phagocytosis instead of directly lysing microbes. To date, many invertebrate sequence resources contain components of the lectin and alternative pathways, in addition to the central molecule C3 (Fujita, 2002; Suzuki et al., 2002; Endo et al., 2003; Clow et al., 2004; Zhu et al., 2005; Castillo et al., 2009; Kimura et al., 2009; Prado-Alvarez et al., 2009; Skjoedt et al., 2010). For the lectin pathway, key initiation molecules including the PRRs mannose-binding lectins (MBL) and ficolins have been characterized (Skjoedt et al., 2010; Baumgarten et al., 2015), in addition to mannose-binding lectin associated serine proteases (MASP), that in vertebrates cleave complement proteins C2 and C4 (Figure 1; Thiel et al., 1997; Matsushita et al., 2000). The alternative pathway serine protease Factor B has also been characterized in several invertebrates (Yoshizaki et al., 2005; Kimura et al., 2009; Prado-Alvarez et al., 2009; Tagawa et al., 2012; Zhong et al., 2012), and in vertebrates plays a role in the formation of the alternative pathway C3 convertase (Sim and Tsiftsoglou, 2004). Functional evidence suggests that complement proteins play a role in interactions with both beneficial and harmful microbes in a variety of invertebrate species (Zhu et al., 2005; Prado-Alvarez et al., 2009; Kvennefors et al., 2010; Collins et al., 2012). However, the dual role of complement in the interaction between symbiosis and disease remains unexplored.

The cnidarian complement system is of interest in the context of both ancestral immunity and symbiosis. Many cnidarians serve as hosts to symbiotic dinoflagellates of the genus *Symbiodinium* (Davy et al., 2012). The role of innate immunity in the onset, maintenance, and breakdown of this relationship is a key area of research as this symbiosis contributes to the overall health of the coral reef ecosystem (Hughes et al., 2003; Weis and Allemand, 2009; Davy et al., 2012). One growing threat to reefs is coral disease, which has increased in both prevalence and frequency due in large part to climate change and anthropogenic stressors (Harvell et al., 2002; Bruno et al., 2003; Sutherland et al., 2004). Despite the role of microbes in both the stability of cnidarian-dinoflagellate symbiosis and in the increasing threat of coral disease, little is known about the cellular mechanisms cnidarians employ to interact with either beneficial or pathogenic microbes. Furthermore, many coral diseases disrupt the stability of cnidarian-dinoflagellate symbiosis, or in some cases specifically target the dinoflagellates (Ben-Haim et al., 2003; Cervino et al., 2004), but the interaction between coral disease and symbiosis remains unknown.

With increased availability of cnidarian sequence resources, complement genes, including C3, Factor B, MASP, MBLs, and ficolins have been characterized in several species (Dishaw et al., 2005; Kimura et al., 2009; Fujito et al., 2010; Kvennefors et al., 2010; Ganot et al., 2011; Shinzato et al., 2011; Hambleton, 2013; Baumgarten et al., 2015; Ocampo et al., 2015). An interesting observation that has emerged from some of these studies is that cnidarians have multiple copies of both C3 and Factor B (Kimura et al., 2009; Ganot et al., 2011; Ocampo et al., 2015). In addition, the sequencing of the *Aiptasia pallida* (also known as *Exaiptasia pallida*, Grajales and Rodriguez, 2014) genome revealed an expansion in fibrinogen domain-containing proteins, many of which are similar to ficolins (CniFLs) (Baumgarten et al., 2015). The multiple copies of complement proteins may allow cnidarians to detect and respond to a greater diversity of MAMPs and differentiate between mutualistic and pathogenic microbes.

Several of these studies have also provided information about the functional role of complement, specifically C3, in cnidarians. C3 gene expression is upregulated in response to challenge with certain bacterial species in the coral *Acropora millepora* (Kvennefors et al., 2010; Brown et al., 2013), which provides some evidence for the traditional role of complement in defense against harmful microbes. Studies have also determined that complement gene expression and proteins are localized to the gastrodermal tissue, the layer where the symbiotic dinoflagellates reside (Kimura et al., 2009; Fujito et al., 2010; Kvennefors et al., 2010; Ganot et al., 2011), suggesting they may play a role in the phagocytosis and entry of symbionts into host cells.

Overall, while there is some evidence to suggest complement proteins participate in cnidarian-dinoflagellate symbiosis and challenge with pathogens, their precise role in these processes remains unknown. In addition, while most studies on cnidarian complement have focused on C3, the functional role of other complement components, such as the serine proteases Factor B and MASP, is largely unexplored. *A. pallida* represents a model system for cnidarian-dinoflagellate symbiosis that can be used to explore the functional role of complement in cnidarians. *A. pallida* is easily maintained in the laboratory in both symbiotic and aposymbiotic states, which makes it a good model for studying the onset of symbiosis and the interaction between symbiosis and disease (Weis et al., 2008). There has been preliminary work to show *A. pallida* C3 protein is localized to the epidermis surrounding the mouth and at the basal surface of the gastrodermal cells, but other complement molecules have not been investigated (Hambleton, 2013). The purpose of this study was to investigate the role of *A. pallida* Factor B and MASP genes in the onset and maintenance of symbiosis and in response to pathogenic bacteria using a qPCR-based approach. In addition, we investigated the existence of multiple Factor B genes in different invertebrates to determine whether these were the result of invertebrate-wide or lineage-specific expansions. Overall, this study contributes to an understanding of the role and evolution of the complement system in basal metazoans.

## Materials and methods

### Complement sequence bioinformatic searches and phylogenetic analysis

Previous studies revealed that cnidarians and other invertebrates have multiple copies of complement genes (Yoshizaki et al., 2005; Kimura et al., 2009; Shinzato et al., 2011; Ocampo et al., 2015), and therefore we were interested in examining whether these were the result of lineage-specific or invertebrate-wide expansions, with a particular focus on cnidarian sequences. A variety of invertebrate genomic and transcriptomic resources were searched for C3, Factor B and MASP sequences. Published invertebrate proteins were obtained from NCBI (http://www.ncbi.nlm.nih.gov) and these sequences were used as queries for the databases listed in Supplementary Table 4. For these searches, invertebrate databases were queried for complement proteins using either BLASTp or tBLASTn searches, with the most closely related annotated protein sequence available. To confirm that these sequences were complement related, they were translated using the ExPASy translate tool (http://web.expasy.org/translate/) and the protein sequence was used as a query in a BLASTp search of the non-redundant (NR) protein database in NCBI (http://blast.ncbi.nlm.nih.gov/Blast.cgi). A sequence was only used for further analysis if the reciprocal top blast hits were to the complement protein of interest. Factor B phylogenetic analysis and a multiple sequence alignment were performed on the protein sequences using the MAFFT v 7.017 plug-in through Geneious using the default settings (Katoh et al., 2002; Drummond et al., 2011). The Factor B sequences were trimmed prior to alignment to contain only the VWA (Von Willabrand Factor type A) and trypsin domains using Pfam annotations due to the variable number of CCP repeats present (http://pfam.sanger.ac.uk; Punta et al., 2012). Sequences that did not span the majority of the alignment or that did not visually align well were removed and regions with gaps were trimmed manually. To determine the best model of protein evolution, ProtTest v 2.4 (Abascal et al., 2005) was run and results were compared using statistical frameworks for all possible substitution matrices and improvements. The best model was determined to be LG+I+G. Maximum likelihood phylogenetic analysis was performed using the PhyML 3.0 web server (http://www.atgc-montpellier.fr/phyml; Guindon and Gascuel, 2003) using the appropriate model of protein evolution and 500 bootstrap replicates. FigTree v1.4 (http://tree.bio.ed.ac.uk/software/figtree/) was used to visualize and midroot the resulting tree with the highest likelihood and to color-code the tree by phylum.

### Animal and algal maintenance

Symbiotic *A. pallida* (VWA population) were maintained at rt under a 12 h light:dark cycle with light intensity of approximately 40 μmol quanta m^−2^ s^−1^. The VWA population is a Weis lab specific strain that has been in existence since 2002 and is composed of clade B containing animals from multiple sources including a local shark tank. To prepare aposymbiotic *A. pallida*, symbiotic animals were chilled at 4°C for 6 h, two times a week for 6 weeks (Muscatine et al., 1991). After the first cold-shock, animals were kept in the dark to avoid re-population with symbionts and cleaned regularly to remove expelled dinoflagellates. All animals were fed brine shrimp (*Artemia*) two times per week. *Symbiodinium minutum* CCMP830 (clade B1) cultures were maintained in F/2 media at 25°C on a 12 h light:dark cycle with a light intensity of 40 μmol quanta m^−2^ s^−1^. New cultures were started each month, in which 50 mL of F/2 media was inoculated with 1 mL of concentrated dinoflagellates. Cultures were grown to a concentration of approximately 10^6^ cells/mL before use in experiments.

### Culturing of *S. marcescens*

*S. marcescens* was cultured either in marine broth (Difco 2216) liquid media or plates at 30°C. As *S. marcescens* is resistant to tetracycline (Cory Krediet, personal communication) the plates were supplemented with 10 μg/ml of tetracycline hydrochloride (Sigma, St. Louis, MO) in order to ensure purity of the culture as previously described (Krediet et al., 2013). Liquid cultures were grown in a shaking incubator at 200 rpm overnight for 18 h before use in experiments.

### Obtaining sequences for *A. pallida* factor B and MASP transcripts

Initially, sequences for Factor B and MASP genes were obtained by a combination of sequence searches in early assemblies of the aposymbiotic CC7 *A. pallida* transcriptome and rapid amplification of cDNA ends (RACE) (Lehnert et al., 2012). All BLAST searches of the transcriptome were performed through Geneious v 5.4.3 (Drummond et al., 2011). Updated versions of the transcriptome were searched for Factor B and MASP sequences as they became available. The sequences presented in this study are from a reassembled version of the raw Illumina sequence reads for accession SRR696721 that were downloaded from the sequence read archive (SRA) entry for the aposymbiotic CC7 transcriptome (http://www.ncbi.nlm.nih.gov/sra/SRX231866) using Trinity (Grabherr et al., 2011). Nucleotide sequences were translated using the ExPASy translate tool (http://web.expasy.org/translate/) and domain structure graphics for the three Factor B and MASP proteins based on Pfam annotations (http://pfam.xfam.org) were created in Domain Graph version 2.0.1 (DOG) (Ren et al., 2009).

### Development of qPCR primers

To confirm that the areas in which qPCR primers were designed did not contain polymorphic sites, each complement transcript was amplified in portions of ~500–1000bp using Geneious v 5.4.3 with the Primer3 module (Supplementary Table 1; Koressaar and Remm, 2007; Drummond et al., 2011; Untergasser et al., 2012) based on sequences in the early assembly of the CC7 aposymbiotic transcriptome (Lehnert et al., 2012). PCRs were performed using the Go Taq Flexi kit (Promega, Madison, WI) with the following protocol: 94°C for 3 min, 35 cycles of 94°C for 45 s, 60°C for 45 s, and 72°C for 1 min, followed by a final extension at 72°C for 10 min. The primer sets for each gene were used on two animals in an effort to capture individual variation that occurs within the lab population. Products were cloned using the pGEM T-easy kit (Promega, Madison, WI) and plasmids isolated using the QiaPrep Spin Miniprep Kit (Qiagen, Valencia, CA). Plasmids were checked for inserts of the correct size using FastDigest EcoRI (Fermentas, Waltham, MA) and sent for Sanger sequencing on the ABI 3730 capillary sequence machine in the Center for Genome Research and Biocomputing (CGRB) at Oregon State University. Based on the sequencing results, conserved regions were targeted for designing qPCR primers in Geneious v.5.4.3 using the Primer3 module that were between 100 and 200 base pairs and that amplified product at an annealing temperature of 60°C (Table 1). PCR products were cloned and sequenced as described above to confirm amplification of the desired product.

**Table 1:** **Primers for qPCR experiments**.

**Gene**	**qPCR Primers**
L10	Forward: 5′- ACG TTT CTG CCG TGG TGT CCC-3′
	Reverse: 5′- CGG GCA GCT TCA AGG GCT TCA-3′
L12	Forward: 5′- ACA TCG CCA AGA CAA TGC GTC C-3′
	Reverse: 5′- GAC GTC ATG GGG CGG CTG TC-3′
PABP	Forward: 5′- GTG CAA GGA GGC GGA CAG CG-3′
	Reverse: 5′- TGG GCT GAT TGC GGG TTG CC-3′
GAPDH	Forward: 5′-TGG TGG CTG CAC CGT CTG GAT-3′
	Reverse: 5′- TGC CAA GGG CGC AAG ACA GT-3′
EF-1 alpha	Forward: 5′-GGC CAA ACC CAG AGA GCA CGC-3′
	Reverse: 5′- GCT CGC TGT ATG GTG GCT CGG-3′
Ap_Bf-1	Forward: 5′-CGG CAA AGG TGT TGC GTG GC-3′
	Reverse: 5′- TGC GGA AAG GCT CCG ATG CG-3′
Ap_Bf-2b	Forward: 5′- AGA GCC GTT TGA GCT TGG CCC-3′
	Reverse: 5′- CCC CAG CCA GCG ACA TAG CC-3′
Ap_MASP	Forward: 5′-GGC GTT CTT GTG GTC ATC CGG G-3′
	Reverse: 5'- CCG CGT CGG GTC ACC ATG C-3′

### Symbiotic state qPCR

qPCR was performed to determine whether there were differences in expression of *A. pallida* Factor B and MASP genes between symbiotic and aposymbiotic anemones. Three aposymbiotic and three symbiotic *A. pallida* were placed in 24-well plates 3 days prior to the start of the experiment and animals were kept in their respective culturing environments. Anemones were starved for 1 week prior to the experiment to avoid brine shrimp contamination. Animals were snap frozen in liquid nitrogen and were kept at −80°C until further use.

### Recolonization experiments

To determine the role of Factor B and MASP in the onset of symbiosis, aposymbiotic *A. pallida* were inoculated with dinoflagellates and gene expression was measured over a 72 h period. Animals were placed in 6-well plates with a total volume of 10 mL of 0.45 μm filtered artificial sea water (FASW) and starved for 4 days prior to the experiment. The morning of the experiment, water changes were performed and the animals were placed in 9 mL of FASW. The treatments used for the experiment were as follows: symbiont+light (SL), no-symbiont+light (NSL), and symbiont+dark (SD). The NSL and SD treatments were included because pilot data indicated that light alone has an impact on complement gene expression (data not shown). The NSL treatment allowed separation of the effects of light and symbionts on complement gene expression and the SD treatment was used to determine the difference between recolonization in the light and dark. For each treatment, animals were sampled at 0, 12, 24, 48, and 72 h post-inoculation (*n* = 3). The SL and SD treatments received 9 mL of FASW, 1 mL of concentrated dinoflagellates, and 40 μl of brine shrimp extract (BSE), which has previously been used in onset of symbiosis studies to promote a feeding response, and therefore symbiont uptake (Schwarz et al., 1999; Weis et al., 2002; Harii et al., 2009). The final dinoflagellate concentration in each well was 2.19 × 10^5^ cells/mL. The NSL treatment received an additional 1 mL of FASW to compensate for the volume of dinoflagellates and 40 μl of BSE. At 24 h post-inoculation, anemones were washed to remove residual symbionts and placed in 10 mL of FASW for the duration of the experiment. At each time point, animals were washed, snap frozen in liquid nitrogen, and kept at −80°C until further use. In this experiment, additional replicates were snap frozen at 24 and 72 h post-inoculation for both SL and SD treatments to quantify relative levels of symbionts within the host.

### Determination of relative symbiont levels for recolonization experiment

Relative symbiont levels were measured during the recolonization experiment to determine whether establishment of symbiosis was successful and to allow correlations to be made between changes in gene expression and symbiont levels within the host. Although, there are several methods used for quantifying *Symbiodinium* density, each technique was developed for particular life-stages (adult vs. larvae), monitoring time frames, or desired quantification outcomes (absolute vs. relative) (summarized in Supplementary Table 2). Many of these techniques lack the sensitivity needed to detect low symbiont abundance at early time points during onset of symbiosis, and those that can detect low symbiont abundance are only applicable to studies in larvae (Supplementary Table 2). Therefore, after unsuccessfully attempting traditional methods, such as hemocytometer counts and Chl-*a* concentrations, the goal was to develop a simple relative qPCR method for symbiont detection in the adult sea anemone *A. pallida*.

DNA was extracted from frozen anemones for aposymbiotic, symbiotic, and recolonized *A. pallida* (*n* = 3, except aposymbiotic group where *n* = 4) using a modified CTAB protocol (Coffroth et al., 1992). Briefly, anemones (average oral disc = 3.84 mm ± 0.14) were homogenized in 2% CTAB using a plastic dounce to break up host tissue. Then, a small amount of 0.6 mm glass beads was added to the homogenate and vortexed for 5 min to break open the cell walls of the CCMP830. To the homogenate, 120 μg/ml of Proteinase K (Thermo Scientific, Waltham, MA) was added and the mixture was incubated for 3 h at 65°C with a brief vortex every 30 min. After incubation, chloroform:isoamyl alcohol (24:1) was added for phase separation. For DNA precipitation, 1 mL of cold 95% ethanol and 200 mM sodium acetate were added to the aqueous phase (top layer) and the solution was incubated overnight at −20°C. The final DNA pellet was washed twice with 70% ethanol, dried, and resuspended in 10 mM Tris Base, pH 8.0.

To estimate relative symbiont density in each anemone, a qPCR assay was designed to compare gene copies of a region within domain 2 of 28S from symbiont nuclear rDNA using clade B1-specific primers that were previously developed by Correa et al. (2009). rDNA genes are useful for detecting *Symbiodinium* clades from complex assemblages *in hospite* (Correa et al., 2009; Arif et al., 2014), however these multicopy genes present challenges for absolute quantification. The gene copy number of 28S in CCMP830 is currently unknown and therefore the underlying assumption of this assay is that 28S gene copy number is the same for each clonal *Symbiodinium* cell. In studies that quantify mixed *Symbiodinium* assemblages within a host, accounting for variation in copy number between symbionts strains is important to obtain accurate results, but it is unnecessary in this study since the levels of just one symbiont type are quantified. Thus, as symbiont numbers increase during early stages of symbiosis establishment, the 28S gene copies will increase proportionally resulting in greater PCR amplification. To account for differences in the *A. pallida* size, qPCR was also performed on the nuclear ribosomal protein L10 (primers in Table 1). The L10 primer set was validated for high PCR efficiency and amplification specificity of *A. pallida* DNA. Each sample was run in triplicate on the MasterCycler RealPlex^4^ real-time PCR machine (Eppendorf, Hamburg, Germany). For each primer set, 15 μl volume qPCR reactions were prepared as follows: 20 ng μl^−1^ genomic DNA, 0.4 μM of the forward and reverse primers, 7.5 μl of 2 × SensiFAST SYBR Hi-ROX master mix (Bioline USA, Boston, MA), and enough molecular grade H_2_O to reach final volume. No-template negative controls were run for each primer pair to check for reagent contamination. The profile for the qPCR run was as follows: 95°C for 2 min for initial denaturing, followed by 40 cycles of 95°C for 5 s, 60°C for 10 s, and 72°C for 20 s. A dissociation curve [95°C for 15 s, 60°C for 15 s, ramping temperature gradient (60–95°C) for 20 min, and 95°C for 15 s] on the final PCR product was performed to confirm single amplicon detection.

The fluorescence baseline and threshold set by the RealPlex^4^ machine was used to calculate C_t_ values, or the first reaction cycle in which fluorescent detection exceeds baseline. Following the Livak 2^ΔΔCt^ method (Livak and Schmittgen, 2001), the symbiont 28S C_t_ from each anemone was normalized to the corresponding anemone reference L10 C_t_. The normalized values (ΔC_t_) were subtracted from the maximum ΔC_t_ (lowest amplification) to obtain the ratio of relative quantities of 28S rDNA per anemone (ΔΔC_t_). All values are expressed as mean relative quantity on the log_2_ scale ± standard error. Relative quantities of 28S rDNA in symbiotic, aposymbiotic and recolonized anemones were compared using a 1-tailed Wilcoxon rank- sum test at a significance threshold of *p* ≤ 0.05.

### Challenge with *S. marcescens*

Prior to the challenge with *S. marcescens*, experiments were performed in which aposymbiotic *A. pallida* were challenged with the individual immune stimulants lipopolysaccharide (LPS) and peptidoglycan (data not shown). However, due to the lack of significant differences in gene expression observed in these studies, we performed the challenge with live bacteria. Aposymbiotic and symbiotic *A. pallida* were placed in 6-well plates approximately 2 weeks prior to the experiment and water changes were performed every other day. Anemones were kept in their respective culturing environments and were fed twice each week as previously described. One week prior to the experiment, water changes were performed with FASW in order to create a more sterile environment for the experiment. Twenty four hours prior to the start of the experiment, the well plates were placed on a shaker table at 65 rpm to acclimate the anemones to the experimental conditions as previously described (Krediet et al., 2013) The symbiotic anemones were kept at rt under ambient lighting on a 12 h light:dark cycle to mimic the natural light conditions. The plates with aposymbiotic anemones were covered to maintain their normal culturing conditions in the dark.

An overnight culture of *S. marcescens* was grown from a single colony and 25 mL was removed and centrifuged for 8 min at 10,000 rpm to pellet the bacteria. The pellet was washed 3 times in autoclaved FASW and the final suspension was diluted to approximately 10^4^ and 10^7^ cells/mL based on OD600 readings. Anemones of each symbiotic state were divided into three treatment groups. To the control treatment, 9 mL of FASW was added to the well. The second treatment was a low concentration of *S. marcescens* in which 9 mL of the 10^4^ cells/mL dilution was added to the well. The third treatment was a high concentration of *S. marcescens* in which 9 mL of the 10^7^ cells/mL dilution was added to the well (*n* = 3 for all treatment types). The anemones were placed on the shaker table at 65 rpm for the duration of the experiment and animals were snap frozen in liquid nitrogen at 0, 24, and 48 h post-challenge with *S. marcescens*. Samples were kept at −80°C until further use.

### RNA extraction and cDNA synthesis

For all animals that were sampled for symbiotic state, recolonization, and *S. marcescens* challenge experiments, total RNA was extracted using a hybrid of the TRIzol Reagent (Life Technologies, Eugene, OR) and RNeasy mini kit (Qiagen, Valencia, CA). Each anemone was homogenized in 500 μL of TRIzol, followed by a centrifugation step to remove debris. A chloroform extraction was performed and the resulting aqueous layer was transferred to a new RNAse-free tube and an equal volume of cold 100% ETOH was added. The samples were then applied to the spin columns from the RNeasy kit and washes were performed per the manufacturer's protocol. RNA was eluted in a final volume of 30 μl of nuclease-free water. The DNA-free kit (Ambion, Waltham, MA) was used to remove contaminating genomic DNA and DNased samples were analyzed using the Nanodrop ND-1000 (Nanodrop Technologies, Wilmington, DE) and resolved on a 1% TBE gel to assess quality and quantity of the RNA obtained. Samples that had a nanodrop 260/230 ratio of less than 1.5 underwent an additional cleanup protocol that involved the addition of 10% 3 M sodium acetate (pH 5.2), RNA-grade glycogen (Fermentas, Waltham, MA) to a final concentration of 0.05–1.0 μg/mL, and 2.5 volumes of 100% ETOH to the sample. Following precipitation overnight at −80°C, samples were centrifuged for 20 min at 9500 rpm (4°C) to pellet the RNA. The pellet was washed with 250 μl of 70% ETOH, centrifuged for 5 min at the same conditions listed above, and dried before resuspending in 20 μl of nuclease-free water. cDNA synthesis was performed with the Superscript III first strand synthesis system (Life Technologies, Eugene, OR) using 750 ng of RNA and the Oligo dT primer. Before use in qPCR, cDNA samples were diluted to a final volume of 50 μl.

### qPCR

For qPCR, samples were run in triplicate, and plates included no-template, no-reverse transcriptase, and no-primer controls, and at least one interplate calibrator (IPC) (except for symbiotic state experiment). The total reaction volume used was 20 μl, which included 7.5 μl of water, 1 μl of each forward and reverse primer (5 μmol), 0.5 μl of the appropriate cDNA template, and 10 μl of Power SYBR Green PCR Master Mix (Applied Biosystems, Waltham, MA). qPCR plates were run using the ABI PRISM 7500 FAST (Applied Biosystems, Waltham, MA) under the standard settings, using the default run protocol with the addition of a melt curve. The Ct values were exported from the machine and imported into GenEx Standard version 5.3.7 (http://www.multid.se). The Ct values were corrected using the interplate calibrator when applicable, qPCR replicates were averaged, and target gene expression was normalized to the reference genes. These values were exported from GenEx and to calculate the ΔΔCt, the normalized value for each sample was subtracted from the average normalized value of a reference sample. The resulting relative quantities on the log_2_ scale were used for statistical analysis. For the symbiotic state comparison, the aposymbiotic samples served as a reference and a 2-tailed Wilcoxon rank-sum test was performed for each gene. For the recolonization experiments, the light and dark data were analyzed separately. For the light data, the NSL sample at each time point was used as a reference, while for the SD samples, time zero was used as the reference sample. Lastly, for the *S. marcescens* challenge, the control sample at each time point was used as a reference. For all of these experiments, a linear model was created, model testing was performed when applicable, and an ANOVA was run followed by Tukey's post-hoc test for pairwise comparisons. All statistical analysis was performed in R version 3.2.1 (RCoreTeam, 2015)

### Determination of qPCR reference genes

Prior to performing qPCR on the genes of interest, reference genes that had stable expression across treatments were determined. The candidate reference genes and samples tested for each experiment are summarized in Supplementary Table 3. qPCR was run on these samples in triplicates for each candidate reference gene, including no-template and no-primer controls, and the resulting Ct values were entered into GeNorm (Vandesompele et al., 2002), Normfinder (Andersen et al., 2004), and Best Keeper (Pfaffl et al., 2004). The results of these programs were compared to determine the three best reference genes for each experiment (Supplementary Table 3). The reference genes from the symbiotic state and recolonization experiments were used for the *S. marcescens* challenge experiment because they were shown in this study to have stable expression between symbiotic states and during challenge with microbes and therefore were deemed the most appropriate.

## Results

### Complement sequence distribution across the invertebrate phyla

**Figure 2:**
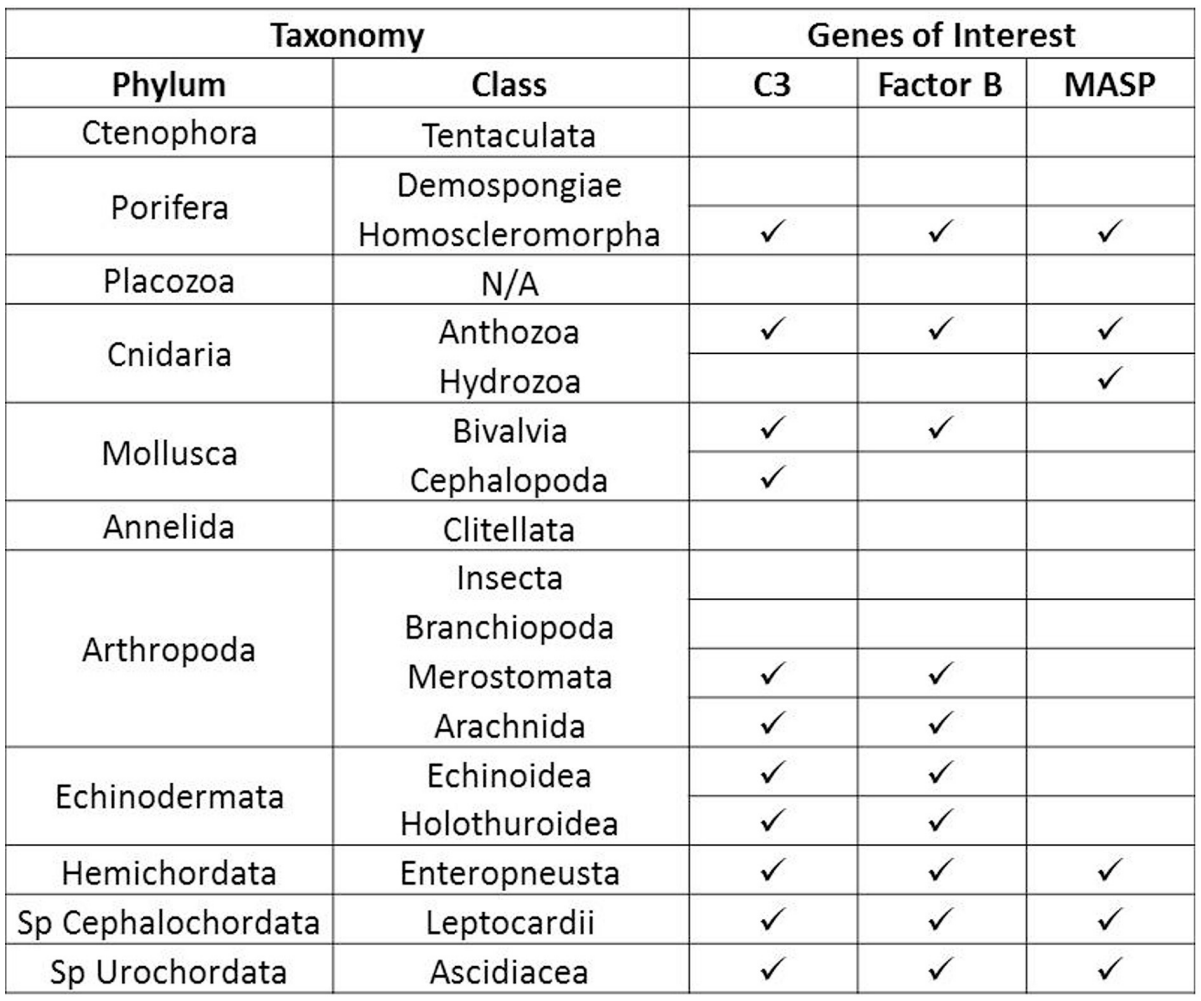
**Summary of Distribution of complement in the invertebrate phyla**. Presence or absence of complement proteins genes C3, Factor B, and MASP is shown for each invertebrate phylum for which sequence databases were searched. A checkmark indicates presence while an empty box indicates absence based on available data.

Sequences for complement proteins were obtained from a variety of invertebrate phyla (Supplementary File 1) and different distribution patterns emerged for each protein. C3 and Factor B were found in the most phyla, but the distribution of MASPs was much more limited (Figure 2). MASPs were only found in four phyla, the Porifera, Cnidaria, Hemichordata, and Chordata (Subphylum Cephalochordata and Urochordata). No evidence of complement genes was identified in the Ctenophora, Placozoa, and Annelida.

Another trend emerging from these data is that complement gene content varied across taxonomic divisions within a phylum. For example in Phylum Cnidaria, members of Class Anthozoa possess C3, Factor B, and MASP, but for the member of Class Hydrozoa examined in this study, only a MASP sequence was found. Class-level differences also occurred within members of Porifera and Mollusca, and at the subphylum level for Arthropoda. However, this is based on currently available data and more invertebrate sequence data will be needed to confirm these trends.

### Phylogenetic analysis of factor B in invertebrates

**Figure 3:**
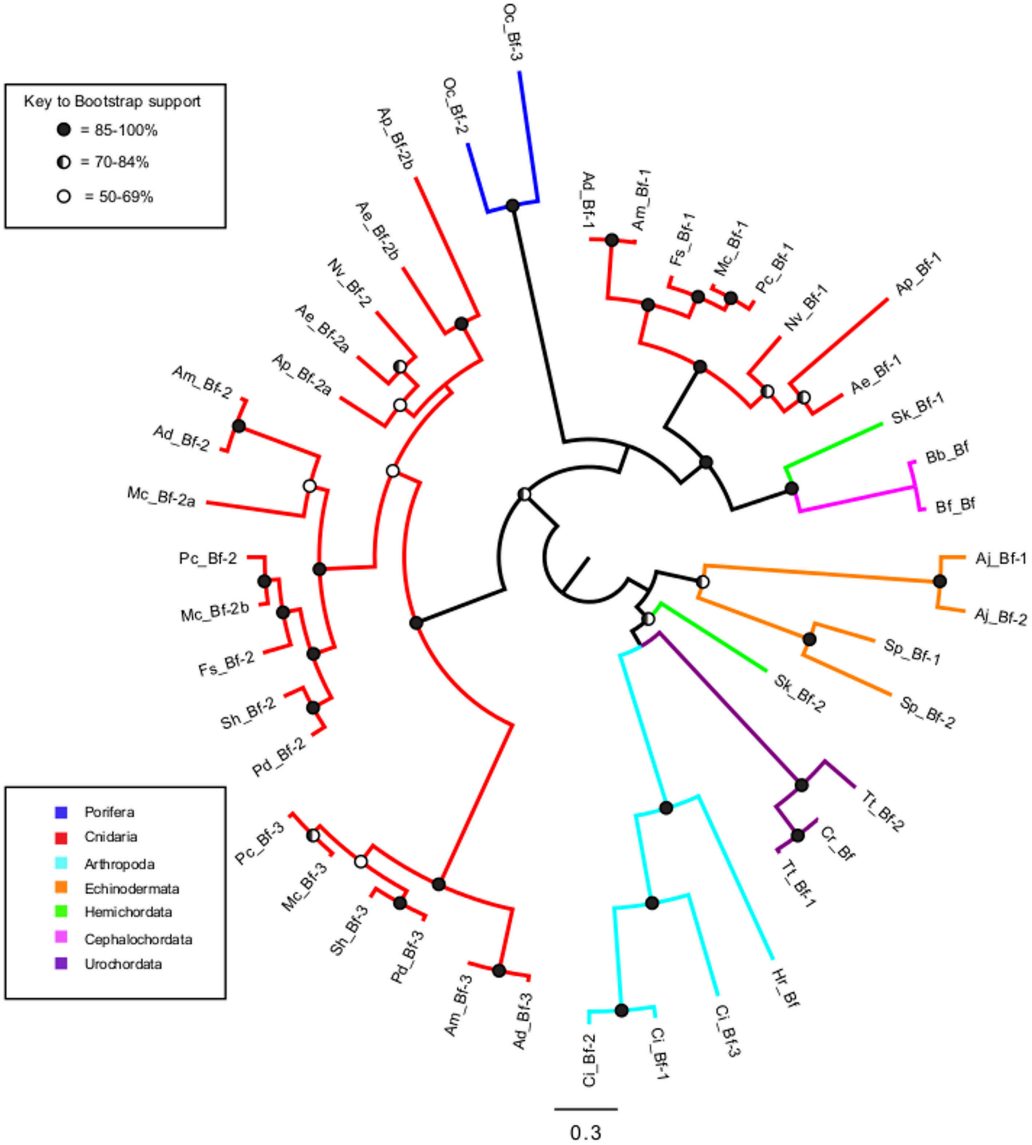
**Maximum likelihood phylogenetic analysis of Factor B proteins in invertebrates**. Sequences are color coded by phylum (subphylum for chordates) and circles at each node represent bootstrap support. Blue, Porifera; Red, Cnidaria; Yellow, Mollusca; Purple, Arthropoda; Orange, Echinodermata; Green, Hemichordata; Magenta, Cephalochordata (subphylum); Aqua, Urochordata (subphylum). Filled circles = 85–100% bootstrap support, half-filled = 70–84% bootstrap support, and open circles = 50–69% bootstrap support.

To determine whether having multiple genes for each complement protein was the result of lineage-specific or invertebrate-wide expansions, maximum likelihood phylogenetic analysis was performed on Factor B sequences obtained from the invertebrate phyla (Figure 3). Many of the shorter sequence fragments obtained from transcriptomic resources were not incorporated into the phylogenetic analysis. The resulting tree revealed that while most sequences grouped in a phylum-specific manner, Cnidaria and Hemichordata sequences split into different groups on the tree. For Cnidaria, there was one group composed of Bf-2 and Bf-3 sequences, while the Bf-1 sequences grouped with high support with the Cephalochordata and one Hemichordata sequence. The two *Saccoglossus kowalevskii* (Hemichordata) sequences appeared in very different places on the tree (Figure 3). Sk_Bf-1 grouped with high support with the Cephalochordata and Cnidaria Bf-1 sequences, while Sk_Bf-2 grouped weakly with the Arthropoda and Urochordata (Figure 3).

### *A. pallida* sequence resources revealed three factor B, and one MASP transcript

**Figure 4:**
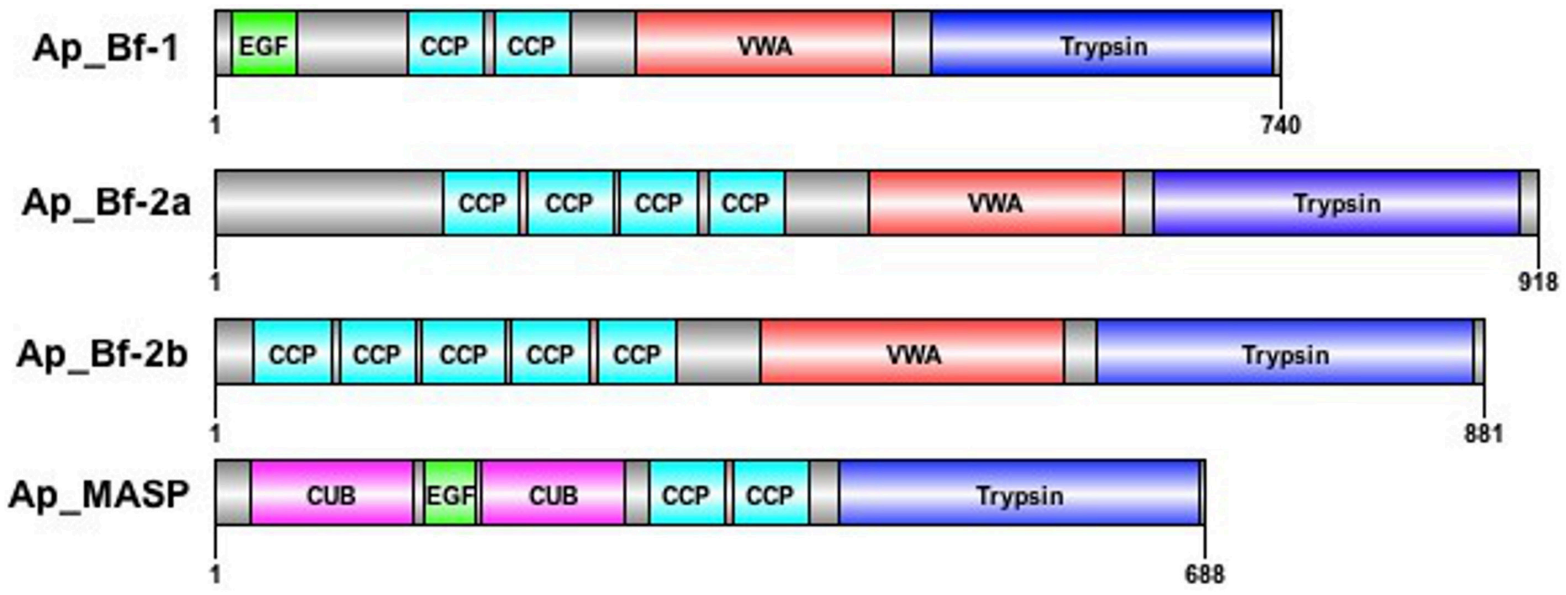
**Domain structures of *A. pallida* Factor B and MASP sequences**. *A. pallida* has three Factor B and one MASP gene. EGF, epidermal growth factor; CCP, complement control protein motif; VWA, Von Willabrand Factor type A domain; Trypsin, serine protease domain; CUB, complement C1r/C1s, Uegf, Bmp1.

Three Factor B and one MASP transcript were obtained through RACE and searches of the *A. pallida* transcriptome (Lehnert et al., 2012; Supplementary File 1). The three Factor B proteins have different domain structures at the N-terminal end (Figure 4). Ap_Bf-1 has an epidermal growth factor (EGF) domain and two complement control protein motifs (CCP), while Ap_Bf-2a and Ap_Bf-2b lack the EGF domain and have 4 and 5 CCP domains respectively. All Factor B protein sequences have a VWA and trypsin domain at the C-terminal end. The Ap_Bf-2a was only recovered in later versions of the *A. pallida* transcriptome and therefore was not available when most of the functional work was conducted. However, it was used in phylogenetic analysis. The domain structure of Ap_MASP is identical to other annotated invertebrate and vertebrate sequences (Figure 4). The three sequences used in the qPCR studies have been submitted to NCBI with the following accession numbers; KU747967 (Ap_Bf-1), KU747968 (Ap_Bf-2b), and KU747969 (Ap_MASP).

### Ap_Bf-2b and Ap_MASP show a trend of repressed expression in symbiotic *A. pallida*

**Figure 5:**
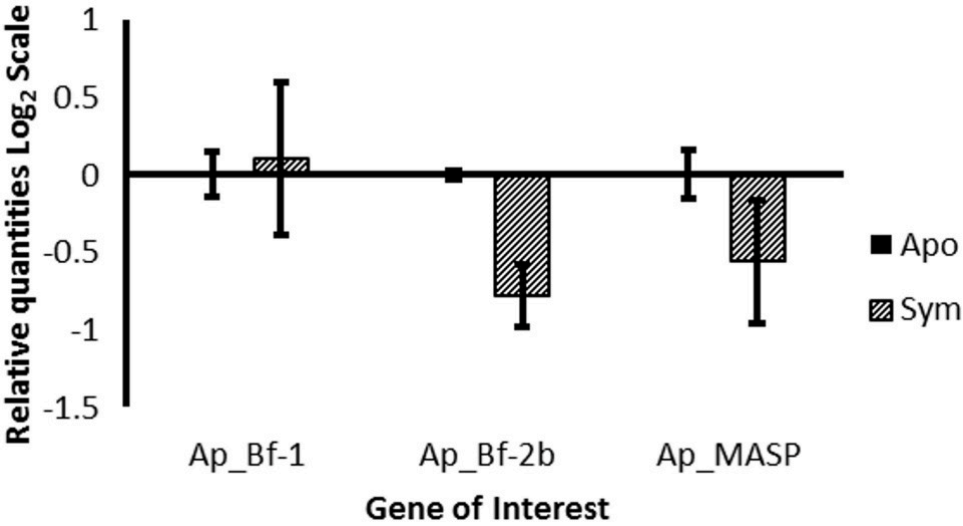
**Expression of Ap_Bf-1, Ap_Bf-2b, and Ap_MASP as a function of symbiotic state**. The relative quantities from qPCR on the log_2_ scale are shown for aposymbiotic organisms (solid bars) and symbiotic organisms (bars with lines). Bars represent means ±SE (*n* = 3).

Comparison of gene expression between aposymbiotic and symbiotic *A. pallida* revealed no significant differences in expression for all three genes, potentially due to the large individual variation that was observed and small samples sizes (Figure 5). However, there is a clear trend of lower expression of both Ap_Bf-2b and Ap_MASP in symbiotic compared to aposymbiotic anemones.

### Recolonization expression patterns vary by gene and light regime

**Figure 6:**
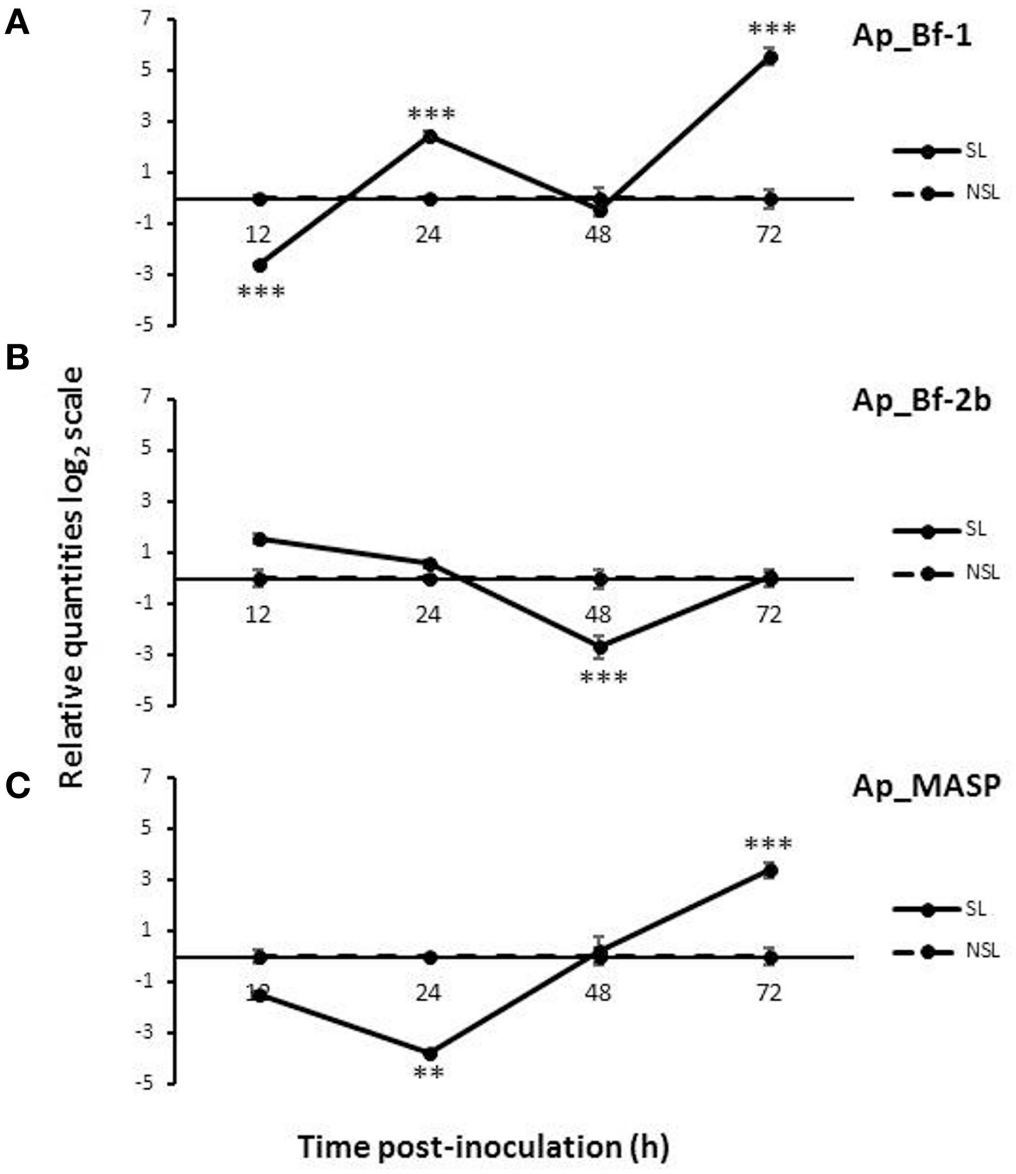
**Expression of Ap_Bf-1, Ap_Bf-2b, and Ap_MASP during the recolonization experiment for symbiont+light (SL) and no symbiont+light (NSL) treatments**. **(A)** Ap_Bf-1, **(B)** Ap_Bf-2b, and **(C)** Ap_MASP). Bars represent means ± SE (*n* = 3) and stars (^*^) represent expression differences that are significantly different between the SL and NSL treatments at a particular time point (ANOVA, Tukey's *post-hoc* test). ^*^p < 0.05, ^**^p < 0.01, ^***^p < 0.001.

The two Factor B and MASP genes each had a distinct expression profile during the course of the 72 h recolonization experiment in response to light and dark conditions. For the light data, Ap_Bf-1 was significantly downregulated for the SL treatment at 12 h post-inoculation with CCMP830 compared to the NSL treatment (*p* < 0.001, ANOVA, Tukey's *post-hoc* test), but at 24 and 72 h post-inoculation was significantly upregulated (*p* < 0.001, ANOVA, Tukey's *post-hoc* test) (Figure 6A). For Ap_Bf-2b, expression was significantly downregulated compared to the NSL treatment at 48 h post-inoculation (*p* < 0.001, ANOVA, Tukey's *post-hoc* test), but returned to levels not significantly different from the NSL control by 72 h post-inoculation (Figure 6B) Ap_MASP shows a similar trend to Ap_Bf-1 in that it was significantly downregulated compared to the NSL at 24 h post-inoculation (*p* < 0.001, ANOVA, Tukey's *post-hoc* test), but was significantly upregulated compared to the control at 72 h post-inoculation (*p* < 0.001, ANOVA, Tukey's HSD) (Figure 6C).

**Figure 7:**
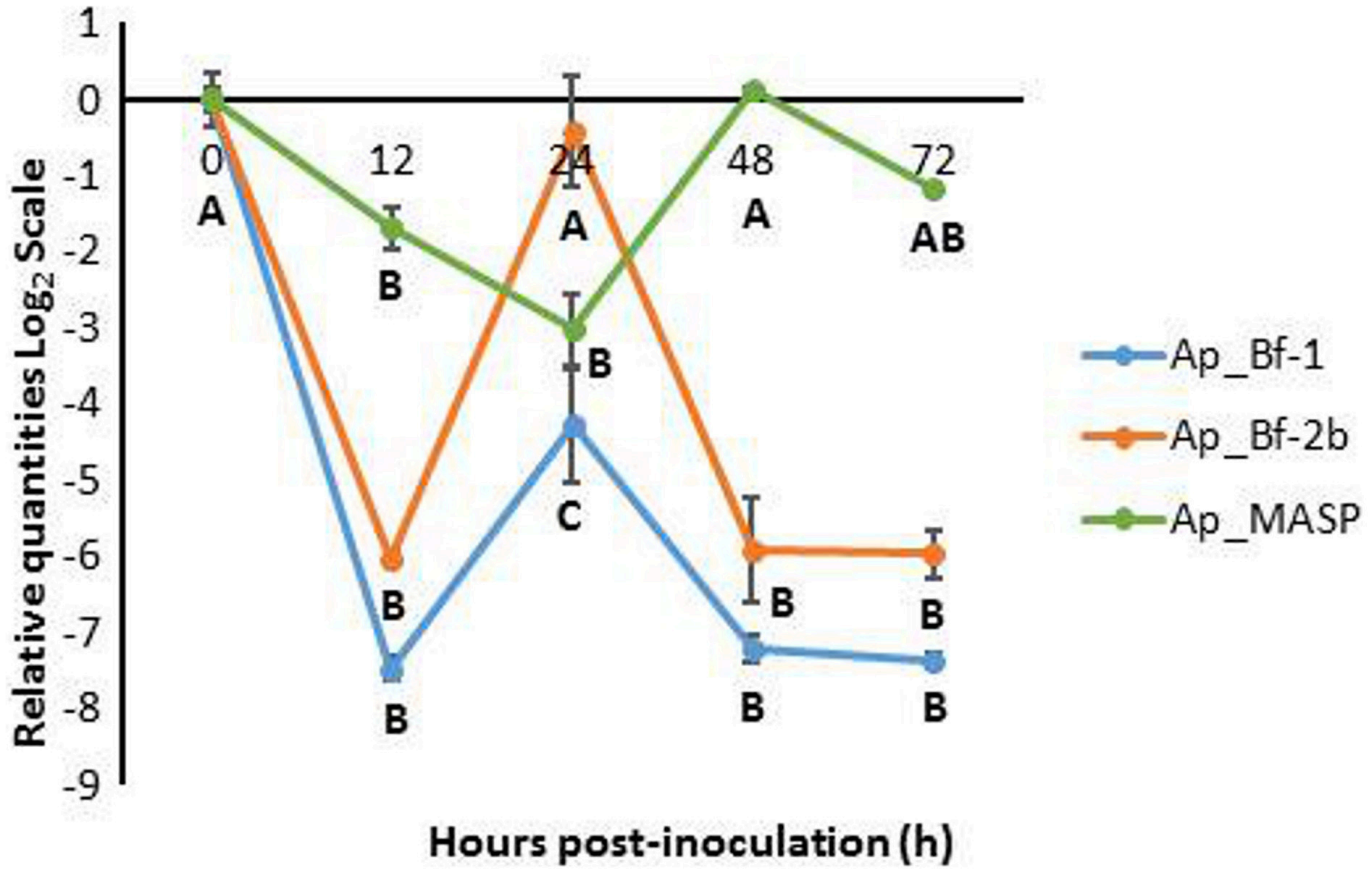
**Expression of Ap_Bf-1, Ap_Bf-2b, and Ap_MASP during the recolonization experiment for symbiont+dark treamtment (SD)**. Bars represent means ± SE (*n* = 3) and letters represent expression differences that are significantly different between time points within each gene (ANOVA, Tukey's *post-hoc* test).

For the SD treatment, all three genes showed consistent downregulation in comparison to the 0 h time point (Figure 7). The expression profiles of Ap_Bf-1 and Ap_Bf-2b were very similar; the only difference is that Ap_Bf-1 shows stronger downregulation at each time point. Both had a peak in expression at 24 h post-inoculation, however for Ap_Bf-1 this was still significantly downregulated compared to the 0 h timepoint (*p* < 0.001, ANOVA, Tukey's *post-hoc* test). Although, Ap_MASP also revealed a trend of suppressed expression in the dark, it showed a slightly different trend than the Factor B genes. Ap_MASP was significantly downregulated at 12 and 24 h post-inoculation (*p* = 0.019 and *p* < 0.001 respectively ANOVA, Tukey's *post-hoc* test), but was not significantly different from the 0 h time point at 48 and 72 h post-inoculation. In addition, the downregulation of Ap_MASP was generally of a much lower magnitude than that observed for Ap_Bf-1 and Ap_Bf-2b.

### Symbiont quantification for recolonization experiment

**Figure 8:**
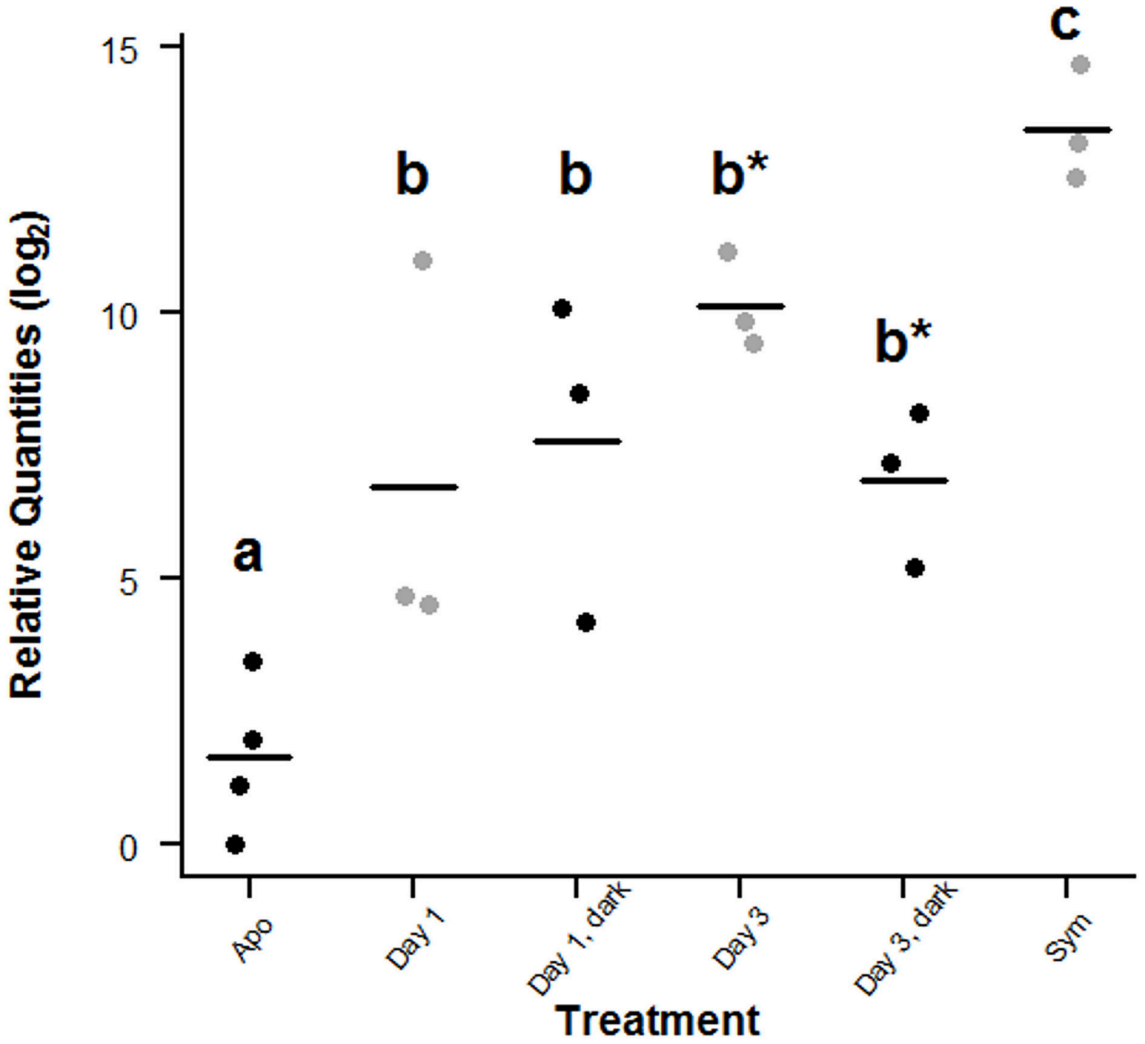
**Comparison of symbiont 28S rDNA in symbiotic, aposymbiotic and recolonized *A. pallida.*** For each treatment group, replicate relative quantities are presented with the mean relative log_2_ quantity indicated by the bar. Points are slightly offset for clarity. Groups that have unique letters (a, b, or c) are significantly different (*p* ≤ 0.05) as indicated by a 1-tailed Wilcoxon rank-sum test. In addition those samples within group B indicated by a star (^*^) are significantly different from each other, but not from the other group B samples.

The qPCR assay revealed differences in the relative amounts of symbiont 28S rDNA between aposymbiotic, symbiotic, and recolonized *A. pallida* (Figure 8). The relative log_2_ quantities of 28S rDNA ranged from 0 to 3.43 in aposymbiotic anemones, indicating that in some cold-shock-treated anemones, residual symbionts were still present, but at very low abundance (Figure 8). This is not uncommon as it is difficult to obtain completely symbiont-free anemones with the cold-shock or chemical treatments (Berner et al., 1993; Matthews et al., 2015). The mean relative log_2_ quantity of the symbiotic anemones was 8.26 times larger than aposymbiotic anemones (*p* = 0.029, Wilcoxon rank-sum test).

During the recolonization experiment, the mean relative log_2_ quantities at 24 and 72 h post-inoculation for the SL treatment were 6.73 ± 2.14 and 10.15 ± 0.52, respectively, both of which were significantly different from the aposymbiotic (*p* = 0.029, Wilcoxon rank-sum test) and symbiotic samples (*p* = 0.05, Wilcoxon rank-sum Test), but not from each other (Figure 8). The lack of significance between 24 and 72 h is likely due to the high variability observed within the 24 h treatment group and small sample sizes. For the SD treatment, the mean relative log_2_quantities at 24 and 72 h post-inoculation were 7.60 ± 1.76 and 6.84 ± 0.86 respectively, both of which were significantly different from aposymbiotic (*p* = 0.029, Wilcoxon rank-sum test) and symbiotic samples (*p* = 0.05, Wilcoxon rank-sum test). As with the results in the light, these data were not significantly different from each other. However, the 72 h SL samples did have significantly higher CCMP830 levels than the 72 h SD samples (*p* = 0.05, Wilcoxon rank-sum test).

### Challenge of aposymbiotic *A. pallida* with *S. marcescens*

**Figure 9:**
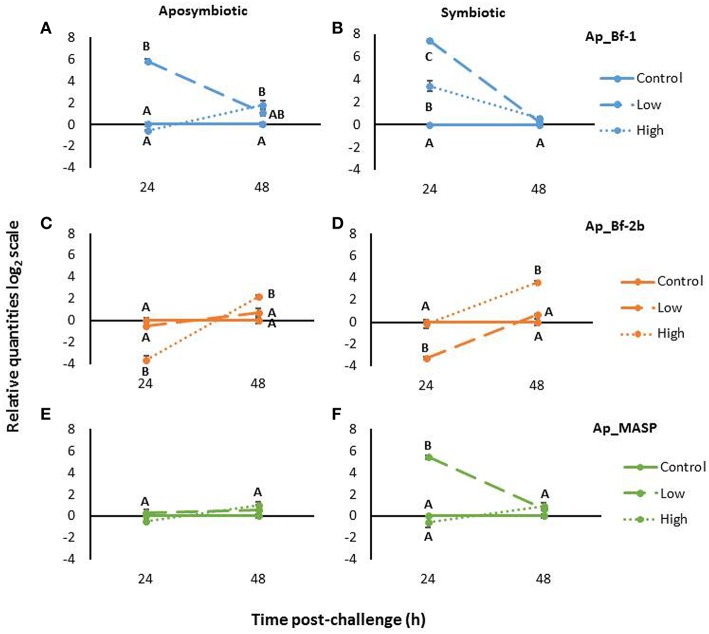
**Expression Ap_Bf-1, Ap_Bf-2b, and Ap_MASP in and aposymbiotic (A,C,E) and symbiotic *A. pallida* (B,D,F) in response to challenge with *S. marcescens* for low bacterial concentrations (10^4^ cells/mL) or high bacterial concentrations (10^7^ cells/mL)**. The relative quantities on the log2 scale are shown and bars represent ±SE (*n* = 3). Letters indicate significant differences in expression within a time point (ANOVA, Tukey's *post-hoc* test).

Aposymbiotic anemones challenged with *S. marcescens* revealed different expression patterns for each complement gene. At 24 h post-challenge, Ap_Bf-1 expression was significantly higher for the low concentration treatment compared to the control and the high concentration treatment (*p* < 0.001, ANOVA, Tukey's *post-hoc* test). The high concentration treatment in contrast was not significantly different than the control at 24 h post-challenge, but was significantly upregulated at 48 h post-challenge (*p* < 0.001, ANOVA, Tukey's *post-hoc* test; Figure 9A).

For the low concentration treatment, Ap_Bf-2b expression levels were not significantly different than controls at either time point. However, for the high concentration treatment, at 24 h post-challenge Ap_Bf-2b expression was significantly lower than in control animals (*p* < 0.001, ANOVA, Tukey's *post-hoc* test; Figure 9C). By 48 h post-challenge, this trend had reversed and the high concentration treatment was significantly higher than the control and low concentration treatment (*p* < 0.001 and *p* = 0.024 respectively, ANOVA, Tukey's *post-hoc* test).

For aposymbiotic *A. pallida*, Ap_MASP showed stable expression in response to challenge with *S. marcescens*. No significant differences were observed for either bacterial concentration at 24 or 48 h post-challenge (Figure 9E).

### Challenge of symbiotic *A. pallida* with *S. marcescens*

Symbiotic *A. pallida* also showed differences in complement gene expression when challenged with either low or high concentrations of *S. marcescens*. Ap_Bf-1 expression was significantly higher than the control at 24 h post-challenge for both the low and high concentrations of bacteria (*p* < 0.001, ANOVA, Tukey's *post-hoc* test; Figure 9B). In addition, the expression of Ap_Bf-1 for anemones challenged with the low concentration treatment was significantly higher than those challenged with the high concentration treatment (*p* < 0.001, ANOVA, Tukey's *post-hoc* test). At 48 h post-challenge, expression of Ap_Bf-1 was not significantly different between the three treatment groups.

Ap_Bf-2b expression for symbiotic animals showed a very different pattern than Ap_Bf-1. At 24 h post-challenge, anemones inoculated with the low concentration treatment had significantly lower expression than control animals (*p* < 0.001, ANOVA, Tukey's *post-hoc* test), but those inoculated with the high concentration treatment were not significantly different from the control animals (Figure 9D). By 48 h post-challenge, expression of Ap_Bf-2b for animals challenged with the high concentration treatment was significantly higher than the low concentration treatment and the control (*p* < 0.001, ANOVA, Tukey's *post-hoc* test), while the low concentration treatment was not significantly different than the control treatment.

Ap_MASP showed a similar trend to Ap_Bf-1 in that at 24 h post-challenge the low concentration treatment showed significantly higher expression than the high concentration treatment and control anemones (*p* < 0.001, ANOVA Tukey's *post-hoc* test), but returned to levels that were not significantly different than the control by 48 h post-challenge (Figure 9F). Ap_MASP expression for the high concentration treatment was not significantly different than the control animals at either 24 or 48 h post-challenge.

### Complement gene expression in response to challenge with *S. marcescens* is different for symbiotic and aposymbiotic *A. pallida*

Another interesting trend to emerge from the *S. marcescens* challenge data was the difference between the responses of symbiotic and aposymbiotic *A. pallida*. First, the symbiotic states differed in their levels of complement expression at time zero. Ap_Bf-1 was significantly higher in symbiotic compared to aposymbiotic anemones (*p* = 0.05, Wilcoxon rank-sum test), while Ap_Bf-2b and Ap_MASP expression was significantly lower in symbiotic anemones (*p* = 0.05, Wilcoxon rank-sum test; Figure S1). There were also expression differences during the duration of the experiment. For Ap_Bf-1, although both symbiotic states overall showed similar trends, in symbiotic animals both the high and low concentration treatments resulted in significantly increased expression at 24 h post-challenge, while for aposymbiotic animals only the low concentration treatment was significantly different than the control (Figures 9A,B). For Ap_Bf-2, aposymbiotic animals only showed significant changes in expression in response to the high concentration treatment, while symbiotic animals showed downregulation in response to the low concentration treatment and upregulation in response to the high concentration treatment (Figures 9C,D). Lastly the most striking difference can be observed for Ap_MASP, in which symbiotic animals showed upregulation in response to the low concentration treatment, while there were no significant differences in expression observed in aposymbiotic animals (Figures 9E,F).

## Discussion

### Bioinformatic searches reveal unique evolutionary patterns of complement genes within each invertebrate phylum

The search for complement genes in the invertebrate phyla revealed several new pieces of information. First, this study represents the first report of Factor B and C3 molecules in Phylum Porifera, from the species *Oscarella carmela*. MASPs and TEPs have previously been reported in members of Class Homoscleromorpha (Riesgo et al., 2014), but this represents the first in-depth look at the complement sequences in this class. This is a surprising discovery given that the genome of *Amphimedon queenslandica*, a member of Class Demospongiae, does not contain C3, Factor B, or MASP (Riesgo et al., 2014). Differences in the presence of complement genes by taxonomic groups within a phylum also occur within Arthropoda and Mollusca (Figure 2). The members of Subphylum Crustacea and Subphylum Hexapoda examined, such as *Daphnia pulex* and *Apis mellifera*, do not have complement genes which is consistent with previous reports from *Drosophila melanogaster* (Adams et al., 2000). In contrast, members of Subphylum Chelicerata have C3 and in some cases, Factor B sequences. Chelicerata is considered basal within Arthropoda, and it has previously been suggested that complement genes were lost in Hexapoda and possibly Crustacea, a hypothesis which is further supported by the data in this study (Sekiguchi et al., 2012; Sekiguchi and Nonaka, 2015). Deeper sampling within these phyla will allow for a better understanding of the evolution of complement genes. These results also indicate that caution should be used when making generalizations about the gene content of a phylum based on sampling of only a few species. Lastly, these differences within a phylum present interesting evolutionary questions about the patterns of complement gene loss and what adaptive pressures have resulted in these events.

Differences in complement gene content between classes within a phylum also occur in Phylum Cnidaria. Members of Class Anthozoa possess at least two C3 genes, 2-3 Factor B genes, and one MASP gene. However, for the hydrozoan, *Hydra vulgaris AEP*, only one MASP gene is present in the transcriptome (Hemmrich et al., 2012). The genome of *Hydra magnipapillata* was also searched and revealed fragments of the MASP gene, but no C3 or Factor B. *Hydra* represents a unique case for complement evolution because it is the only organism in this study that has MASP, but not C3 or Factor B. At this time, there have been no studies performed on the *Hydra* MASP, but how this protein functions in the absence of other complement components warrants further investigation.

Overall, sequences of complement genes identified in invertebrates indicates that the evolutionary history of C3, Factor B, and MASP is complex and that there have been multiple occurrences of either gene loss or gene duplication that warrant further investigation. The evolutionary pressures that have led to these events are currently unknown, but a testable hypothesis is that they are linked to lifestyle and the diversity of the microbial community interacting with the animal host.

### Phylogenetic analysis of factor B reveals that multiple complement genes appear to be the result of lineage-specific rather than invertebrate-wide evolutionary events

Maximum likelihood phylogenetic analysis of Factor B proteins in invertebrates revealed that most sequences grouped in a phylum-specific manner (Figure 3). This suggests that the multiple copies of complement genes are likely the result of lineage-specific rather than invertebrate-wide expansions, but these may occur at either the phylum level or at higher taxonomic divisions. The exception was that the Cnidaria and Hemichordata sequences each fall within two different clades on the tree. The cnidarian Bf-2 and Bf-3 sequences form a group on the tree with high support, but the Bf-1 sequences form a highly supported group with the cephalochordate and one of the hemichordate sequences. The other Hemichordata sequence grouped with the Arthorpoda and Urochordata. As most of these groupings are highly supported, this suggests that Factor B may have a complex evolutionary history. Future, work describing the functional divergence of Factor B genes in cnidarians may provide more insight into these potential gene duplication events.

Another interesting observation about Factor B is the difference between corals and anemones. Coral sequences form three distinct Bf-1, Bf-2, and Bf-3 groups on the tree, while anemones lack sequences that group with the coral Bf-3 group. However, support for placement of the anemone sequences was weak, therefore further work needs to be performed to determine the phylogenetic relationship of anthozoan Factor B sequences. In addition, *A. pallida* and *Anthopleura elegantissima* have three Factor B sequences, while *Nematostella vectensis* has only two. From the limited taxa surveyed, it is difficult to fully understand the evolutionary history of Factor B in cnidarians, but as more sequence data become available, future studies can further investigate these patterns. Based on the current data, a testable hypothesis is that the number of Factor B genes may be related to symbiotic state as all the symbiotic cnidarians in this study had three Factor B genes, while the non-symbiotic anemone *N. vectensis* has only two.

Overall, from the phylogenetic analysis of Factor B it can be concluded that the presence of multiple copies of Factor B in some phyla is generally the result of lineage-specific and not invertebrate-wide expansions. Therefore, potential gene duplication and subsequent functional divergence or gene loss events may have occurred in response to the lifestyle or environmental conditions that a particular taxonomic group experienced through evolutionary time.

### Reduced Ap_Bf-2b and Ap_MASP expression in the symbiotic state suggests immunomodulation of the host immune system in order to maintain symbiosis

In the original comparison between symbiotic and aposymbiotic anemones, there were no significant differences in expression, but there was a trend of repressed Ap_Bf-2b and Ap_MASP expression in symbiotic animals compared to aposymbiotic animals (Figure 5). Furthermore, a comparison of the 0 h time point between symbiotic and aposymbiotic animals for the *S. marcescens* challenge showed the same trends obtained during the original symbiotic state comparison with the exception that in this experiment, all three genes were significantly differentially expressed between symbiotic states (Figure S1). Together these results suggest that Ap_Bf-1 is upregulated in symbiotic anemones, while Ap_Bf-2b and Ap_MASP are downregulated in symbiotic animals. However, as the magnitude of these differences varied across experiments, higher sample sizes could help to confirm these results. Overall, these data suggest that the presence of symbionts modulates expression of host complement system genes.

The idea that microbes can alter the functioning of the host immune system has been shown in many systems, including cnidarian-dinoflagellate symbiosis. For example, previous work revealed that stimulation with LPS resulted in the production of higher levels of nitric oxide (used as a proxy for an immune response) in aposymbiotic compared to symbiotic *A. pallida* (Detournay et al., 2012). In addition, the CniFL genes, which encode ficolin-like proteins that may serve as PRRs overall had greater expression in aposymbiotic than symbiotic *A. pallida* (Baumgarten et al., 2015). Together, these data suggest that the presence of symbionts suppresses some aspects of the host immune response, which is similar to the results obtained for Ap_Bf-2b and Ap_MASP. Furthermore, the results obtained for Factor B in this study are similar to those for C3 in the anemone *Anemonia viridis*, where one C3 isoform had repressed expression in symbiotic animals, while the other showed no difference between symbiotic states (Ganot et al., 2011). While the suppression of certain immune pathways may be important for tolerance of mutualistic microbes within host cells, it is likely that this may also influence the interactions between cnidarians and pathogenic microbes they encounter, perhaps making them susceptible to secondary infections.

Immune modulation of the host also occurs in the symbiosis between the squid *Euprymna scolopes* and the luminescent bacterium *Vibrio fischeri* and has specifically been documented for the complement system. C3 expression was detected in naïve or cured host hemocytes, but not in those from individuals with fully colonized light organs, suggesting that the complement response in symbiotic organisms is suppressed (Collins et al., 2012). In the context of these studies, it can be hypothesized that the repressed expression of Ap_Bf-2b and Ap_MASP in the symbiotic state allows for maintenance of the symbiosis and aids in preventing the host from mounting an immune response against the symbionts. This is also supported by the downregulation of *A. pallida* CnidFLs genes in the symbiotic state, which potentially serve as upstream PRRs that activate the complement system (Baumgarten et al., 2015). Ap_Bf-1 in contrast, may directly interact with symbionts and promote a stable partnership. Overall, the results from the symbiotic state comparison support immunomodulation of the host by the symbiont and provide the first line of evidence for the functional divergence of Ap_Bf-1 and Ap_Bf-2b.

### Recolonization experiments reveal upregulation of Ap_Bf-1 and Ap_MASP, but downregulation of Ap_Bf-2b in the light at the onset of symbiosis

The recolonization experiment revealed significant upregulation of Ap_Bf-1 and Ap_MASP by 72 h post-inoculation in the light compared to the control NSL treatment (Figures 6A,C). Symbiont quantification revealed a trend of increasing symbiont levels in the host between 24 and 72 h post-inoculation (Figure 8). Since anemones were washed at 24 h post-inoculation, increased symbiont densities at 72 h could be a result of symbiont proliferation within the host tissue, which suggests successful uptake and establishment of symbiosis. Therefore the corresponding increase in Ap_Bf-1 and Ap_MASP expression at the same time points may indicate a role for these proteins in the establishment of a successful symbiosis. Specifically, as they are upregulated when symbionts are proliferating in host tissue they may be involved in entry of symbionts into host cells. Ap_Bf-1 was also significantly upregulated by 24 h post-inoculation which also suggests it may play a role in the initial recognition and phagocytosis of symbionts.

Ap_Bf-2b was downregulated at 48 h post-inoculation compared to the NSL control treatment, but returned to levels that were not significantly different from the control by 72 h post-inoculation (Figure 6B). Later time points could establish a more conclusive trend in expression, but the downregulation of Ap_Bf-2b is consistent with the data from the symbiotic state comparison that revealed that the presence of symbionts leads to repressed Ap_Bf-2b expression. Future experiments that study the transition from the onset to a fully established partnership would be useful to further explore this trend.

### Complement gene expression is suppressed during inoculation with symbionts in the dark

The DS treatment resulted in downregulation of all three complement genes for the majority of the 72 h recolonization experiment (Figure 7). The overall trend of Ap_Bf-1 and Ap_Bf-2b expression was identical. Ap_MASP showed a similar trend to the Factor B genes, but the lowest expression was offset by 24 h, and reached a low point at 48 h post-inoculation. Ap_MASP also showed much less dramatic downregulation than either of the Factor B genes compared to the 0 h time point.

The observed repression of complement gene expression in the dark suggests that light is an important signal for expression of complement genes. This is not a surprising result because light and symbiosis with photosynthetic *Symbiodinium* are tightly coupled and light-induced expression has also been observed for sym32, a cell adhesion protein previously shown to be involved in symbiosis in the anemones *A. elegantissima* and *A. viridis* (Schwarz, 2002; Ganot et al., 2011). The observed repression of complement gene expression in the dark may also be related to the fact that symbiont levels in host decreased slightly (not significantly) between 24 and 72 h post-inoculation indicating that CCMP830 in the dark were not proliferating within the host as was suggested by the light data (Figure 8). Therefore complement gene expression may be repressed when conditions are not favorable for a successful partnership.

### Challenge with a low concentration of *S. marcescens* results in opposing responses for Ap_Bf-1 and Ap_MASP compared to Ap_Bf-2b

At 24 h post-challenge with *S. marcescens*, Ap_Bf-1 was significantly upregulated in both aposymbiotic and symbiotic animals exposed to low concentrations of bacteria (Figures 9A,B). The same trend was observed for Ap_MASP in symbiotic animals only (Figures 9E,F). By 48 h post-challenge, this difference is no longer significantly different than the control for either symbiotic state, which suggests that Ap_Bf-1 and Ap_MASP are more responsive to low concentrations of *S. marcescens* at early time points. Since both Factor B and MASP act upstream of C3 cleavage in vertebrates, it is possible that by 48 h post-challenge, events downstream of C3 cleavage are occurring which may include activation of other innate immune pathways. The similarities in the trends observed for Ap_Bf-1 and Ap_MASP suggest that these proteins are functioning together. In mice, MASP-1 was shown to cleave Factor D and was therefore essential for alternative pathway activation (Takahashi et al., 2010). Therefore, although no Factor D homologs have been characterized in cnidarians or other invertebrates, a testable hypothesis is that Ap_MASP contributes to activation of Ap_Bf-1, perhaps by direct cleavage or indirectly by activation of another serine protease.

In contrast to Ap_Bf-1 and Ap_MASP, Ap_Bf-2b is downregulated in response to the low concentration treatment at 24 h post-challenge in symbiotic animals, but returns to control levels by 48 h post-challenge (Figure 9D). For aposymbiotic animals there are no significant differences between the control and low concentration treatment. These results indicate that Ap_Bf-2b is not involved in the response against low concentrations of *S. marcescens* and expression is instead repressed. The opposing expression patterns observed for Ap_Bf-1 and Ap_Bf-2b for the low concentration treatment suggests functional divergence for these two proteins. This overall trend of upregulation of Ap_Bf-1 and downregulation of Ap_Bf-2b observed in the *S. marcescens* challenge is also similar to the trend obtained for the recolonization experiment at the onset of symbiosis. Together these data provide further evidence for functional diversification in *A. pallida* Factor B genes and that Ap_Bf-1 and Ap_MASP, but not Ap_Bf-2b is involved in response to either harmful or beneficial microbes.

### Challenge with a high concentration of *S. marcescens* induces less dramatic changes in gene expression for Ap_Bf-1 and Ap_MASP than the low concentration treatment

An unexpected result was that for Ap_Bf-1 in aposymbiotic animals and Ap_MASP in both symbiotic and aposymbiotic anemones, there were fewer significant differences in expression initiated by the high compared to the low concentration treatments. This indicates that these genes are less responsive to high concentrations of *S. marcescens* at the time points examined in this study (Figure 9). It may be that the high concentration resulted in a peak in expression of Ap_Bf-1 and Ap_MASP in earlier time points that were not measured in this study.

In contrast, Ap_Bf-2b levels showed a more complex trend in expression for the high concentration treatment and also showed differences between symbiotic state (Figures 9C,D). For symbiotic anemones, Ap_Bf-2b expression at 24 h post-challenge was not significantly different from the control, but at 48 h post-challenge was significantly higher than the control. For aposymbiotic organisms, Ap_Bf-2b was significantly lower than the control at 24 h post-challenge, but significantly higher by 48 h post-challenge. Therefore, these data suggest that Ap_Bf-2b expression is not activated until 48 h post-challenge, and is even repressed at early time points in aposymbiotic animals. Together the results provide further evidence for functional divergence between the two *A. pallida* Factor B genes and that Ap_Bf-2b may be important in later stages of defense against pathogens.

### Differences in complement gene expression between symbiotic and aposymbiotic anemones suggests that symbionts modulate the host immune response

Although symbiotic and aposymbiotic animals showed many similarities in their response to *S. marcescens* challenge, there were also marked differences. First, at time zero, Ap_Bf-1 expression was significantly higher in symbiotic than aposymbiotic animals, but for Ap_Bf-2b and Ap_MASP the pattern was the reverse (Figure S1). These data match the trends obtained from the symbiotic state comparison (Figure 5). During the duration of the experiment, Ap_Bf-1 showed a significant increase in expression at 24 h post-challenge for both the low and high concentrations of *S. marcescens*, while for aposymbiotic animals there were no significant changes for the high bacterial concentration. Similarly, for Ap_MASP, the symbiotic animals had increased expression for the low bacterial concentration at 24 h post-challenge, but for aposymbiotic organisms there was no significant change for either concentration. Together, these data suggest for certain treatments, Ap_Bf-1 and Ap_MASP expression is less responsive, or in some cases not responsive at all in aposymbiotic anemones during immune challenge. This is in contrast to other innate immunity studies in *A. pallida* that indicate the immune response of symbiotic organisms is repressed (Detournay et al., 2012). A testable hypothesis that would explain the results observed in this study is that the complement response for symbiotic organisms is influenced by the presence of both pathogenic bacteria and the interaction between *S. minutum* and *S. marcescens*. Recent histological work revealed that *S. marcescens* leads to loss of symbionts in *A. pallida* (Krediet et al., 2014), so although the symbiotic animals in this experiment showed no visible signs of bleaching, this process may have begun at the cellular level. Future work that examines the influence of bleaching on complement gene expression and the interaction between *Symbiodinium* and *S. marcescens* could provide further information about the results presented in this study for symbiotic organisms.

Another testable hypothesis is that the presence of symbionts alters the microbiome associated with *A. pallida* and therefore influences the infection dynamics of *S. marcescens*. *S. marcescens* is traditionally considered a human pathogen, which enters the marine environment through sewage waste (Sutherland et al., 2010), and in *Acropora palmata* colonization and utilization of the coral mucus is an essential step of the infection process (Krediet et al., 2013). Specifically, some commensal bacteria of *A. palmata* have been shown to inhibit the production of enzymes involved in breakdown of the coral mucus and biofilm formation by *S. marcescens*, thereby inhibiting its growth (Alagely et al., 2011; Krediet et al., 2012). Therefore, the presence of dinoflagellates may influence the bacterial community associated with *A. pallida*, which in turn modulates interactions between the natural microbiota and *S. marcescens*.

### Overall conclusions

The results from this study give a holistic view of the role of the complement system in cnidarian-dinoflagellate symbiosis and immune challenge in the anemone *A. pallida*. The results from both the recolonization experiment and challenge with *S. marcescens* reveal that Ap_Bf-1 and Ap_MASP are upregulated in response to inoculation with either harmful or beneficial microbes, suggesting that they play a role in both onset of symbiosis and the immune response. However, this upregulation was only seen in the light for the recolonization experiment and was a more dramatic response in symbiotic animals with a low concentration of bacteria for the *S. marcescens* challenge. Together these results suggest that a variety of factors including light, concentration of microbe, and symbiotic state influence complement gene expression.

Ap_Bf-2b in contrast to Ap_Bf-1 and Ap_MASP shows a more complex expression profile to different types of microbes. In the recolonization experiment, Ap_Bf-2b expression was downregulated in the SL treatment compared to the NSL treatment at 48 h post-inoculation, but returned to levels not significantly different from the control by the end of the experiment. In response to the *S. marcescens* challenge, Ap_Bf-2b showed the opposite expression profile to that of Ap_Bf-1 and Ap_MASP. The low concentration resulted in either significant downregulation or no change in expression at 24 h post-challenge and the high concentration resulted in upregulation at 48 h post-inoculation. These opposing expression profiles suggest functional divergence between Ap_Bf-1 and Ap_Bf-2b. They may be the result of an ancient duplication and have since evolved distinct functions in the regulation of the *A. pallida* microbial community. The downregulation of Ap_Bf-2b observed in these experiments and the downregulation in symbiotic animals compared to aposymbiotic animals overall suggests that the presence of beneficial or harmful microbes suppresses Ap_Bf-2b expression. Therefore the role that Ap_Bf-2b plays in the *A. pallida* immune response remains unclear.

Results from the sequence searches and phylogenetic analysis demonstrated that there were often differences between members of the same phylum in complement gene content, suggesting that these genes have been gained or lost many times during the course of evolution. Specifically, the pattern of Factor B evolution in cnidarians revealed interesting trends that indicate potential differences between corals and anemones, which warrants further investigation. The results from the phylogenetic analysis also align with the expression profiles obtained in this study for Ap_Bf-1 and Ap_Bf-2b. Ap_Bf-1 groups with other invertebrate sequences and appears to play a conserved role in response to microbes, whereas Ap_Bf-2b is within a cnidarian specific group and is downregulated in the presence of microbes. Future work that investigates functional divergence of cnidarian Factor B proteins may offer insight into their evolutionary patterns.

## Author contributions

AP, SK and VW conceived the experiments presented in this manuscript. AP and SK developed the qPCR analysis pipeline and performed the qPCR experiments for symbiotic state comparison and recolonization experiment. Analysis of complement gene expression data for these experiments was performed by AP. SK performed qPCR experiments for the symbiont quantification for the recolonization experiment and performed data analysis. AP performed the *S. marcescens* challenge and associated data analysis. AP completed all sequence searches and phylogenetic analysis for complement sequences in invertebrates. AP and SK made significant contributions to the manuscript. All authors approved the final manuscript.

## Funding

Funding for this work was provided by NSF grant number IOS091973 and by the Oregon State University Department of Integrative Biology.

### Conflict of interest statement

The authors declare that the research was conducted in the absence of any commercial or financial relationships that could be construed as a potential conflict of interest.
