# Supplementary material for: The Role of Complement in Cnidarian-Dinoflagellate Symbiosis and Immune Challenge in the Sea Anemone Aiptasia pallida
Source: Front Microbiol. 2016 Apr 22;7:519. doi: 10.3389/fmicb.2016.00519 (PMC4840205; doi:10.3389/fmicb.2016.00519)
Supplement: Supplementary file 1 [file Table1.DOCX]

Supplementary Table 1. Primers for RACE and polymorphism checks of Factor B and MASP

| Gene | Primers |
| --- | --- |
| Ap_Bf-1 3’ RACE | Outer 969: 5’-GAA GCG AGC AAT ACA TTA GGG-3’  Inner 1049: 5’-CTT TTA AAA CTA CAG AAC AAG GCA AAA CTG -3’ |
| Ap_Bf-1 5’ RACE | Outer: 255: 5’-GAA TTC GAA ACG CCA AAC TC-3’  Inner 174: 5’-GAA CTG TCA AAT ACG AAG ACA AGA TCA AG-3’ |
| Ap_Bf-1 Poly | F1375: 5’ GGG GAA TCA AAC ACA GGA AG-3’  R240: 5’ ATC TGC GGT CCT TGT ATT CG-3’  R395: 5’ ATC CTC ATG GCG AGG TAC AC-3’ |
| Ap_Bf-2b Poly | F1: 5’ TCG CGG TCG AGT TGC AGG TG 3  R1: 5’ CCC CAG CCA GCG ACA TAG CC-3’ |
| Ap_MASP Poly | F4: 5’- ACG TCG CGG TGG TCC ATT GTG-3’  R4: 5’- TCG TCG GCA CTG ACT CAG CC-3’ |
