# Supplementary material for: The Role of Complement in Cnidarian-Dinoflagellate Symbiosis and Immune Challenge in the Sea Anemone Aiptasia pallida
Source: Front Microbiol. 2016 Apr 22;7:519. doi: 10.3389/fmicb.2016.00519 (PMC4840205; doi:10.3389/fmicb.2016.00519)
Supplement: Supplementary file 2 [file Table2.DOCX]

**Supplementary Table 2**: Summary of *Symbiodinium* quantification techniques and application for studying the onset of cnidarian-dinoflagellate symbiosis.

| Technique | Absolute numbers | Low symbiont density | Temporal scale | Life Stage | Selected studies | Attempted in this study |
| --- | --- | --- | --- | --- | --- | --- |
| Hemocytometer  cell counts | ✓ | 🗶 | Days to months | Adult | (Coffroth et al., 2001; Coffroth et al., 2010; Hill et al., 2014) | ✓ |
| Flow cytometry  cell counts | ✓ | ✓ | 1 month | Adult | (Hambleton et al., 2014) |  |
| Cell counts in wholemounts | ✓ | ✓ | Hours to days | Larvae | (Weis et al., 2001; Weis et al., 2002; Rodriguez-Lanetty et al., 2006; Harii et al., 2009) |  |
| Stereo-microscope: total autofluorescence of symbionts | 🗶 | 🗶 | Within a week | Adult | (Hambleton et al., 2014) |  |
| Confocal microscopy: total autofluorescence of symbionts | 🗶 | ✓ | Within a day | Adult (tentacle) | (Detournay et al., 2012) |  |
| Chl-*a* content | 🗶 | 🗶 | 2 days | Adult | (Berner et al., 1993) | ✓ |
| FISH ^ | ✓ | ✓ | N/A | Adult | N/A |  |
| HTS ^ | ✓ | ✓ | N/A | Adult | N/A |  |
| qPCR ^ | ✓  (if absolute) | ✓ | N/A | Adult | N/A | ✓ |

^ These quantification methods have been applied to studies that describe proportion of different *Symbiodinium* types within a host (Fitt et al., 2000; Loram et al., 2007; Correa et al., 2009; Mayfield et al., 2009; Mieog et al., 2009; Fay and Weber, 2012; Byler et al., 2013; Arif et al., 2014; Hill et al., 2014; McIlroy et al., 2014; Quigley et al., 2014) or loss of symbionts during a natural or laboratory-induced bleaching event (Jones, 1997; Warner et al., 1999; Belda-Baillie et al., 2002; Venn et al., 2006; Ganot et al., 2011; Cunning and Baker, 2013; Hill et al., 2014), but have not yet been applied to onset of symbiosis studies.

FISH = Fluorescence *in situ* hybridization; HTS = high-throughput sequencing.

References:

Arif, C., Daniels, C., Bayer, T., Banguera-Hinestroza, E., Barbrook, A., Howe, C.J., LaJeunesse, T.C., and Voolstra, C.R. (2014). Assessing *Symbiodinium* diversity in scleractinian corals via next-generation sequencing-based genotyping of the ITS2 rDNA region. *Mol. Ecol.* 23**,** 4418-4433. doi: 10.1111/mec.12869.

Belda-Baillie, C.A., Baillie, B.K., and Maruyama, T. (2002). Specificity of a model cnidarian-dinoflagellate symbiosis. *Biol. Bull.* 202**,** 74-85.

Berner, T., Baghdasarian, G., and Muscatine, L. (1993). Repopulation of a sea anemone with symbiotic dinoflagellates: analysis by *in vivo* fluorescence. *J. Exp. Mar. Biol. Ecol.* 170**,** 145-158.

Byler, K.A., Carmi-Veal, M., Fine, M., and Goulet, T.L. (2013). Multiple symbiont acquisition strategies as an adaptive mechanism in the coral *Stylophora pistillata*. *PLoS One* 8**,** e59596.

Coffroth, M.A., Poland, D.M., Petrou, E.L., Brazeau, D.A., and Holmberg, J.C. (2010). Environmental symbiont acquisition may not be the solution to warming seas for reef-building corals. *PLoS One* 5**,** e13258.

Coffroth, M.A., Santos, S.R., and Goulet, T.L. (2001). Early ontogenetic expression of specificity in a cnidarian-algal symbiosis. *Mar. Ecol. Prog. Ser.* 222**,** 85-96.

Correa, A.M., McDonald, M.D., and Baker, A.C. (2009). Development of clade-specific *Symbiodinium* primers for quantitative PCR (qPCR) and their application to detecting clade D symbionts in Caribbean corals. *Mar Biol* 156**,** 2403-2411.

Cunning, R., and Baker, A.C. (2013). Excess algal symbionts increase the susceptibility of reef corals to bleaching. *Nature Climate Change* 3**,** 259-262.

Detournay, O., Schnitzler, C.E., Poole, A., and Weis, V.M. (2012). Regulation of cnidarian–dinoflagellate mutualisms: evidence that activation of a host TGFβ innate immune pathway promotes tolerance of the symbiont. *Dev. Comp. Immunol.* 38**,** 525-537.

Fay, S.A., and Weber, M.X. (2012). The occurrence of mixed infections of *Symbiodinium* (Dinoflagellata) within individual hosts. *J. Phycol.* 48**,** 1306-1316.

Fitt, W., McFarland, F., Warner, M., and Chilcoat, G. (2000). Seasonal patterns of tissue biomass and densities of symbiotic dinoflagellates in reef corals and relation to coral bleaching. *Limnol. Oceanogr.* 45**,** 677-685.

Ganot, P., Moya, A., Magnone, V., Allemand, D., Furla, P., and Sabourault, C. (2011). Adaptations to endosymbiosis in a cnidarian-dinoflagellate association: differential gene expression and specific gene duplications. *PLoS genetics* 7**,** e1002187.

Hambleton, E.A., Guse, A., and Pringle, J.R. (2014). Similar specificities of symbiont uptake by adults and larvae in an anemone model system for coral biology. *J. Exp. Biol.* 217**,** 1613-1619. doi: 10.1242/jeb.095679.

Harii, S., Yasuda, N., Rodriguez-Lanetty, M., Irie, T., and Hidaka, M. (2009). Onset of symbiosis and distribution patterns of symbiotic dinoflagellates in the larvae of scleractinian corals. *Mar Biol* 156**,** 1203-1212.

Hill, R., Fernance, C., Wilkinson, S.P., Davy, S.K., and Scott, A. (2014). Symbiont shuffling during thermal bleaching and recovery in the sea anemone *Entacmaea quadricolor*. *Mar. Biol.* 161**,** 2931-2937.

Jones, R.J. (1997). Changes in zooxanthellar densities and chlorophyll concentrations in corals during and after a bleaching event. *Mar. Ecol. Prog. Ser.* 158**,** 51-59.

Loram, J.E., Boonham, N., O'Toole, P., Trapido-Rosenthal, H.G., and Douglas, A.E. (2007). Molecular quantification of symbiotic dinoflagellate algae of the genus *Symbiodinium*. *Biol. Bull.* 212**,** 259-268.

Mayfield, A.B., Hirst, M.B., and Gates, R.D. (2009). Gene expression normalization in a dual‐compartment system: a real‐time quantitative polymerase chain reaction protocol for symbiotic anthozoans. *Molecular Ecology Resources* 9**,** 462-470.

McIlroy, S., Smith, G., and Geller, J. (2014). FISH-Flow: a quantitative molecular approach for describing mixed clade communities of *Symbiodinium*. *Coral Reefs* 33**,** 157-167.

Mieog, J.C., van Oppen, M.J., Berkelmans, R., Stam, W.T., and Olsen, J.L. (2009). Quantification of algal endosymbionts (*Symbiodinium*) in coral tissue using real‐time PCR. *Molecular Ecology Resources* 9**,** 74-82.

Quigley, K.M., Davies, S.W., Kenkel, C.D., Willis, B.L., Matz, M.V., and Bay, L.K. (2014). Deep-sequencing method for quantifying background abundances of *Symbiodinium* types: exploring the rare *Symbiodinium* biosphere in reef-building corals. *PLoS One* 9**,** e94297.

Rodriguez-Lanetty, M., Wood-Charlson, E.M., Hollingsworth, L.L., Krupp, D.A., and Weis, V.M. (2006). Temporal and spatial infection dynamics indicate recognition events in the early hours of a dinoflagellate/coral symbiosis. *Mar. Biol.* 149**,** 713-719.

Venn, A.A., Wilson, M.A., Trapido-Rosenthal, H.G., Keely, B.J., and Douglas, A.E. (2006). The impact of coral bleaching on the pigment profile of the symbiotic alga, *Symbiodinium*. *Plant, Cell Environ.* 29**,** 2133-2142.

Warner, M.E., Fitt, W.K., and Schmidt, G.W. (1999). Damage to photosystem II in symbiotic dinoflagellates: a determinant of coral bleaching. *Proceedings of the National Academy of Sciences* 96**,** 8007-8012.

Weis, V.M., Reynolds, W.S., and Krupp, D.A. (2001). Host-symbiont specificity during onset of symbiosis between the dinoflagellates *Symbiodinium* spp. and planula larvae of the scleractinian coral *Fungia scutaria*. *Coral Reefs* 20**,** 301-308.

Weis, V.M., Verde, E.A., Pribyl, A., and Schwarz, J.A. (2002). Aspects of the larval biology of the sea anemones Anthopleura elegantissima and A. artemisia. *Invertebrate Biology* 121**,** 190-201.
