# Supplementary material for: The Role of Complement in Cnidarian-Dinoflagellate Symbiosis and Immune Challenge in the Sea Anemone Aiptasia pallida
Source: Front Microbiol. 2016 Apr 22;7:519. doi: 10.3389/fmicb.2016.00519 (PMC4840205; doi:10.3389/fmicb.2016.00519)
Supplement: Supplementary file 3 [file Table3.DOCX]

**Supplementary Table 3**: Determination of the most stable reference genes for qPCR experiments

| **Experiment** | **Treatments tested** | **Candidate Genes Tested** | **Best Reference Genes** |
| --- | --- | --- | --- |
| Symbiotic State | Apo vs Sym | L10, L12, PABP, Actin, GAPDH, and EF-1α | L10, L12, PABP |
| Recolonization | 3hr vs 72hr | L10, L12, PABP, Actin, GAPDH, and EF-1α | L10, L12, PABP |
| *S. marcescens* | N/A | N/A | L10, L12, PABP |
