# Supplementary material for: The Role of Complement in Cnidarian-Dinoflagellate Symbiosis and Immune Challenge in the Sea Anemone Aiptasia pallida
Source: Front Microbiol. 2016 Apr 22;7:519. doi: 10.3389/fmicb.2016.00519 (PMC4840205; doi:10.3389/fmicb.2016.00519)
Supplement: Supplementary file 4 [file Table4.DOCX]

**Supplementary Table 4: Invertebrate resources searched for complement sequences**

| **Phylum** | **Class** | **Organism** | **Resource Searched** | **Publication** |
| --- | --- | --- | --- | --- |
| Ctenophora | Tentaculata | *Mnemiopsis leidyi* | *Mnemiopsis* Genome Project Portal (http://research.nhgri.nih.gov/mnemiopsis/blast/) | (Ryan et al., 2013; Moreland et al., 2014) |
| Porifera | Demospongiae | *Amphimedon queenslandica* | Ensembl  (http://metazoa.ensembl.org/Amphimedon_queenslandica) | (Srivastava et al., 2010; Kersey et al., 2014) |
|  | Homoscleromorpha | *Oscarella carmela* | Compagen  http://www.compagen.org/index.html | (Nichols et al., 2012) |
| Placozoa | N/A | *Trichoplax adherens* | JGI Genome Portal  (http://genome.jgipsf.org/Triad1/Triad1.home.html) | (Srivastava et al., 2008) |
| Cnidaria | Anthozoa | *Acropora digitifera* | OIST Marine Genomic Unit Genome Browser  (http://marinegenomics.oist.jp/genomes/gallery) | (Shinzato et al., 2011) |
|  |  | *Acropora millepora* | http://www.bio.utexas.edu/research/matz_lab/matzlab/Data.html | (Moya et al., 2012) |
|  |  | *Anthopleura elegantissima* | http://people.oregonstate.edu/~meyere/data.html | (Kitchen et al., 2015) |
|  |  | *Aiptasia pallida* | http://pringlelab.stanford.edu/projects.html | (Lehnert et al., 2012) |
|  |  | *Fungia scutaria* | http://people.oregonstate.edu/~meyere/data.html | (Kitchen et al., 2015) |
|  |  | *Montastrea cavernosa* | http://people.oregonstate.edu/~meyere/data.html | (Kitchen et al., 2015) |
|  |  | *Nematostella vectensis* | JGI Genome Portal  (http://genome.jgi-psf.org/Nemve1/Nemve1.home.html) | (Putnam et al., 2007; Kimura et al., 2009) |
|  |  | *Platygyra carnosus* | PcarnBase  (http://www.comp.hkbu.edu.hk/~db/PcarnBase/#&panel1-3) | (Sun et al., 2013) |
|  |  | *Pocillopora damicornis* | PdamBase  (http://cnidarians.bu.edu/PdamBase/cgi-bin/index.cgi) | (Traylor-Knowles et al., 2011) |
|  |  | *Seriatopora hystrix* | http://people.oregonstate.edu/~meyere/data.html | (Kitchen et al., 2015) |
|  |  | *Swiftea exserta* | N/A | (Dishaw et al., 2005) |
|  | Hydrozoa | *Hydra vulgaris AEP* | Compagen  http://www.compagen.org/index.html | (Hemmrich and Bosch, 2008; Hemmrich et al., 2012) |
| Mollusca | Gastropoda | *Lottia gigantea* | JGI Genome Portal  (http://genome.jgi.doe.gov/Lotgi1/Lotgi1.info.html) | (Simakov et al., 2012) |
|  | Bivalvia | *Crassostrea gigas* | Oyster DB:  (http://oysterdb.cn/home.html) | (Zhang et al., 2012) |
|  |  | *Venerupis decussatus* | N/A | (Prado-Alvarez et al., 2009) |
|  | Cephalopoda | *Euprymna scolopes* | N/A | (Castillo et al., 2009) |
| Annelida | Clitellata | *Capitella teleta* | JGI Genome Portal  http://genome.jgi.doe.gov/Capca1/Capca1.home.html | (Simakov et al., 2012) |
|  |  | *Helobdella robusta* | JGI Genome Portal  http://genome.jgi.doe.gov/Helro1/Helro1.home.html | (Simakov et al., 2012) |
| Arthropoda | Insecta | *Apis melifera* | BeeBase  (http://hymenopteragenome.org/beebase/?q=apis_blast) | (Consortium, 2006; Munoz-Torres et al., 2011) |
|  | Branchiopoda | *Daphnia pulex* | JGI Genome Portal  (http://genome.jgi.doe.gov/Dappu1/Dappu1.home.html) | (Colbourne et al., 2011) |
|  | Merostomata | *Carcinoscorpius rotundicauda* | N/A | (Zhu et al., 2005) |
|  |  | *Tachypleus tridentatus* | N/A | (Ariki et al., 2008) |
|  | Arachnida | *Metaseiulus occidentalis* | N/A (obtained from NCBI) | N/A |
| Echinodermata | Echinoidea | *Strongylocentrotus purpuratus* | SpBAse:  http://www.spbase.org/SpBase/ | (Al-Sharif et al., 1998; Smith et al., 1998; Sodergren et al., 2006; Cameron et al., 2009) |
|  | Holothuroidea | *Apostichopus japonicus* | N/A | (Zhou et al., 2011; Zhong et al., 2012) |
| Hemichordata | Enteropneusta | *Saccoglossus kowalevskii* | Metazome v3.0:  (http://www.metazome.net/search.php?org=Org_Skowalevskii) | N/A |
| Cephalochordata (Subphylum) | Leptocardii | *Brachiostoma belcheri* | N/A | (Suzuki et al., 2002; He et al., 2008; Wang et al., 2009) |
|  | Leptocardii | *Branchiostoma floridae* | JGI Genome Portal:  JGI (http://genome.jgi-psf.org/Brafl1/Brafl1.home.html) | (Putnam et al., 2008) |
| Urochordata (Subphylum) | Ascidiacea | *Ciona intestinalis* | JGI Genome portal  ( http://genome.jgi-psf.org/Cioin2/Cioin2.home.html) | (Dehal et al., 2002; Marino et al., 2002) |
|  |  | *Halocynthia roretzi* | N/A | (Endo et al., 2003) |

References:

Al-Sharif, W.Z., Sunyer, J.O., Lambris, J.D., and Smith, L.C. (1998). Sea urchin coelomocytes specifically express a homologue of the complement component C3. *The Journal of Immunology* 160**,** 2983-2997.

Ariki, S., Takahara, S., Shibata, T., Fukuoka, T., Ozaki, A., Endo, Y., Fujita, T., Koshiba, T., and Kawabata, S.I. (2008). Factor C Acts as a Lipopolysaccharide-Responsive C3 Convertase in Horseshoe Crab Complement Activation. *J Immunol* 181**,** 7994-8001.

Cameron, R.A., Samanta, M., Yuan, A., He, D., and Davidson, E. (2009). SpBase: the sea urchin genome database and web site. *Nucleic Acids Res* 37**,** D750-D754.

Castillo, M.G., Goodson, M.S., and McFall-Ngai, M. (2009). Identification and molecular characterization of a complement C3 molecule in a lophotrochozoan, the Hawaiian bobtail squid *Euprymna scolopes*. *Dev Comp Immunol* 33**,** 69-76. doi: Doi 10.1016/J.Dci.2008.07.013.

Colbourne, J.K., Pfrender, M.E., Gilbert, D., Thomas, W.K., Tucker, A., Oakley, T.H., Tokishita, S., Aerts, A., Arnold, G.J., and Basu, M.K. (2011). The ecoresponsive genome of *Daphnia pulex*. *Science* 331**,** 555-561.

Consortium, H.G.S. (2006). Insights into social insects from the genome of the honeybee Apis mellifera. *Nature* 443**,** 931.

Dehal, P., Satou, Y., Campbell, R.K., Chapman, J., Degnan, B., De Tomaso, A., Davidson, B., Di Gregorio, A., Gelpke, M., and Goodstein, D.M. (2002). The draft genome of *Ciona intestinalis*: insights into chordate and vertebrate origins. *Science* 298**,** 2157-2167.

Dishaw, L.J., Smith, S.L., and Bigger, C.H. (2005). Characterization of a C3-like cDNA in a coral: phylogenetic implications. *Immunogenetics* 57**,** 535-548. doi: Doi 10.1007/S00251-005-0005-1.

Endo, Y., Nonaka, M., Saiga, H., Kakinuma, Y., Matsushita, A., Takahashi, M., Matsushita, M., and Fujita, T. (2003). Origin of mannose-binding lectin-associated serine protease MASP-1 and MASP-3 involved in the lectin complement pathway traced back to the invertebrate, amphioxus. *J Immunol* 170**,** 4701-4707.

He, Y.N., Tang, B., Zhang, S.C., Liu, Z.H., Zhao, B.S., and Chen, L.L. (2008). Molecular and immunochemical demonstration of a novel member of Bf/C2 homolog in amphioxus *Branchiostoma belcheri*: Implications for involvement of hepatic cecum in acute phase response. *Fish Shellfish Immun* 24**,** 768-778. doi: Doi 10.1016/J.Fsi.2008.03.004.

Hemmrich, G., and Bosch, T. (2008). Compagen, a comparative genomics platform for early branching metazoan animals reveals early origins of of genes regulating stem cell differentiation *BioEssays* 30**,** 1010-1018.

Hemmrich, G., Khalturin, K., Boehm, A.-M., Puchert, M., Anton-Erxleben, F., Wittlieb, J., Klostermeier, U.C., Rosenstiel, P., Oberg, H.-H., and Domazet-Lošo, T. (2012). Molecular signatures of the three stem cell lineages in *Hydra* and the emergence of stem cell function at the base of multicellularity. *Mol Biol Evol* 29**,** 3267-3280.

Kersey, P.J., Allen, J.E., Christensen, M., Davis, P., Falin, L.J., Grabmueller, C., Hughes, D.S.T., Humphrey, J., Kerhornou, A., and Khobova, J. (2014). Ensembl Genomes 2013: scaling up access to genome-wide data. *Nucleic Acids Res* 42**,** D546-D552.

Kimura, A., Sakaguchi, E., and Nonaka, M. (2009). Multi-component complement system of Cnidaria: C3, Bf, and MASP genes expressed in the endodermal tissues of a sea anemone, *Nematostella vectensis*. *Immunobiology* 214**,** 165-178. doi: Doi 10.1016/J.Imbio.2009.01.003.

Kitchen, S.A., Crowder, C.M., Poole, A.Z., Weis, V.M., and Meyer, E. (2015). De Novo Assembly and Characterization of Four Anthozoan (Phylum Cnidaria) Transcriptomes. *G3: Genes| Genomes| Genetics* 5**,** 2441-2452.

Lehnert, E.M., Burriesci, M.S., and Pringle, J.R. (2012). Developing the anemone *Aiptasia* as a tractable model for cnidarian-dinoflagellate symbiosis: the transcriptome of aposymbiotic *A. pallida*. *BMC genomics* 13**,** 271.

Marino, R., Kimura, Y., De Santis, R., Lambris, J.D., and Pinto, M.R. (2002). Complement in urochordates: cloning and characterization of two C3-like genes in the ascidian Ciona intestinalis. *Immunogenetics* 53**,** 1055-1064. doi: Doi 10.1007/A00251-001-0421-9.

Moreland, R.T., Nguyen, A.-D., Ryan, J.F., Schnitzler, C.E., Koch, B.J., Siewert, K., Wolfsberg, T.G., and Baxevanis, A.D. (2014). A customized Web portal for the genome of the ctenophore *Mnemiopsis leidyi*. *BMC genomics* 15**,** 316.

Moya, A., Huisman, L., Ball, E., Hayward, D., Grasso, L., Chua, C., Woo, H., Gattuso, J.P., Forêt, S., and Miller, D. (2012). Whole transcriptome analysis of the coral *Acropora millepora* reveals complex responses to CO2‐driven acidification during the initiation of calcification. *Mol Ecol* 21**,** 2440-2454.

Munoz-Torres, M.C., Reese, J.T., Childers, C.P., Bennett, A.K., Sundaram, J.P., Childs, K.L., Anzola, J.M., Milshina, N., and Elsik, C.G. (2011). Hymenoptera Genome Database: integrated community resources for insect species of the order Hymenoptera. *Nucleic Acids Res* 39**,** D658-D662.

Nichols, S.A., Roberts, B.W., Richter, D.J., Fairclough, S.R., and King, N. (2012). Origin of metazoan cadherin diversity and the antiquity of the classical cadherin/β-catenin complex. *P Natl Acad Sci USA* 109**,** 13046-13051.

Prado-Alvarez, M., Rotllant, J., Gestal, C., Novoa, B., and Figueras, A. (2009). Characterization of a C3 and a factor B-like in the carpet-shell clam, *Ruditapes decussatus*. *Fish Shellfish Immun* 26**,** 305-315. doi: Doi 10.1016/J.Fsi.2008.11.015.

Putnam, N.H., Butts, T., Ferrier, D.E., Furlong, R.F., Hellsten, U., Kawashima, T., Robinson-Rechavi, M., Shoguchi, E., Terry, A., and Yu, J.-K. (2008). The amphioxus genome and the evolution of the chordate karyotype. *Nature* 453**,** 1064-1071.

Putnam, N.H., Srivastava, M., Hellsten, U., Dirks, B., Chapman, J., Salamov, A., Terry, A., Shapiro, H., Lindquist, E., Kapitonov, V.V., Jurka, J., Genikhovich, G., Grigoriev, I.V., Lucas, S.M., Steele, R.E., Finnerty, J.R., Technau, U., Martindale, M.Q., and Rokhsar, D.S. (2007). Sea anemone genome reveals ancestral eumetazoan gene repertoire and genomic organization. *Science* 317**,** 86-94. doi: Doi 10.1126/Science.1139158.

Ryan, J.F., Pang, K., Schnitzler, C.E., Nguyen, A.-D., Moreland, R.T., Simmons, D.K., Koch, B.J., Francis, W.R., Havlak, P., and Smith, S.A. (2013). The genome of the ctenophore *Mnemiopsis leidyi* and its implications for cell type evolution. *Science* 342**,** 1242592.

Shinzato, C., Shoguchi, E., Kawashima, T., Hamada, M., Hisata, K., Tanaka, M., Fujie, M., Fujiwara, M., Koyanagi, R., and Ikuta, T. (2011). Using the *Acropora digitifera* genome to understand coral responses to environmental change. *Nature* 476**,** 320-323.

Simakov, O., Marletaz, F., Cho, S.-J., Edsinger-Gonzales, E., Havlak, P., Hellsten, U., Kuo, D.-H., Larsson, T., Lv, J., and Arendt, D. (2012). Insights into bilaterian evolution from three spiralian genomes. *Nature*.

Smith, L.C., Shih, C.S., and Dachenhausen, S.G. (1998). Coelomocytes express SpBf, a homologue of factor B, the second component in the sea urchin complement system. *J Immunol* 161**,** 6784-6793.

Sodergren, E., Weinstock, G.M., Davidson, E.H., Cameron, R.A., Gibbs, R.A., Angerer, R.C., Angerer, L.M., Arnone, M.I., Burgess, D.R., and Burke, R.D. (2006). The genome of the sea urchin *Strongylocentrotus purpuratus*. *Science* 314**,** 941-952.

Srivastava, M., Begovic, E., Chapman, J., Putnam, N.H., Hellsten, U., Kawashima, T., Kuo, A., Mitros, T., Salamov, A., and Carpenter, M.L. (2008). The *Trichoplax* genome and the nature of placozoans. *Nature* 454**,** 955-960.

Srivastava, M., Simakov, O., Chapman, J., Fahey, B., Gauthier, M.E., Mitros, T., Richards, G.S., Conaco, C., Dacre, M., and Hellsten, U. (2010). The *Amphimedon queenslandica* genome and the evolution of animal complexity. *Nature* 466**,** 720-726.

Sun, J., Chen, Q., Lun, J.C., Xu, J., and Qiu, J.-W. (2013). PcarnBase: Development of a transcriptomic database for the brain coral *Platygyra carnosus*. *Marine Biotechnology* 15**,** 244-251.

Suzuki, M.M., Satoh, N., and Nonaka, M. (2002). C6-like and C3-like molecules from the cephalochordate, amphioxus, suggest a cytolytic complement system in invertebrates. *J Mol Evol* 54**,** 671-679. doi: Doi 10.1007/S00239-001-0068-Z.

Traylor-Knowles, N., Granger, B., Lubinski, T., Parikh, J., Garamszegi, S., Xia, Y., Marto, J., Kaufman, L., and Finnerty, J. (2011). Production of a reference transcriptome and transcriptomic database (PocilloporaBase) for the cauliflower coral, *Pocillopora damicornis*. *BMC genomics* 12**,** 585.

Wang, G., Zhang, S., and Wang, Z. (2009). Responses of alternative complement expression to challenge with different combinations of *Vibrio anguillarum*, *Escherichia coli* and *Staphylococcus aureus*: Evidence for specific immune priming in amphioxus *Branchiostoma belcheri*. *Fish Shellfish Immun* 26**,** 33-39.

Zhang, G., Fang, X., Guo, X., Li, L., Luo, R., Xu, F., Yang, P., Zhang, L., Wang, X., and Qi, H. (2012). The oyster genome reveals stress adaptation and complexity of shell formation. *Nature* 490**,** 49-54.

Zhong, L., Zhang, F., and Chang, Y. (2012). Gene cloning and function analysis of complement B factor-2 of *Apostichopus japonicus*. *Fish Shellfish Immun* 33**,** 504-513.

Zhou, Z., Sun, D., Yang, A., Dong, Y., Chen, Z., Wang, X., Guan, X., Jiang, B., and Wang, B. (2011). Molecular characterization and expression analysis of a complement component 3 in the sea cucumber *Apostichopus japonicus*. *Fish Shellfish Immun* 31**,** 540-547.

Zhu, Y., Thangamani, S., Ho, B., and Ding, J.L. (2005). The ancient origin of the complement system. *Embo J* 24**,** 382-394. doi: Doi 10.1038/Sj.Emboj.7600533.
