# Supplementary material for: The Role of Complement in Cnidarian-Dinoflagellate Symbiosis and Immune Challenge in the Sea Anemone Aiptasia pallida
Source: Front Microbiol. 2016 Apr 22;7:519. doi: 10.3389/fmicb.2016.00519 (PMC4840205; doi:10.3389/fmicb.2016.00519)
Supplement: Supplementary file 6 [file FigureS1.PDF]

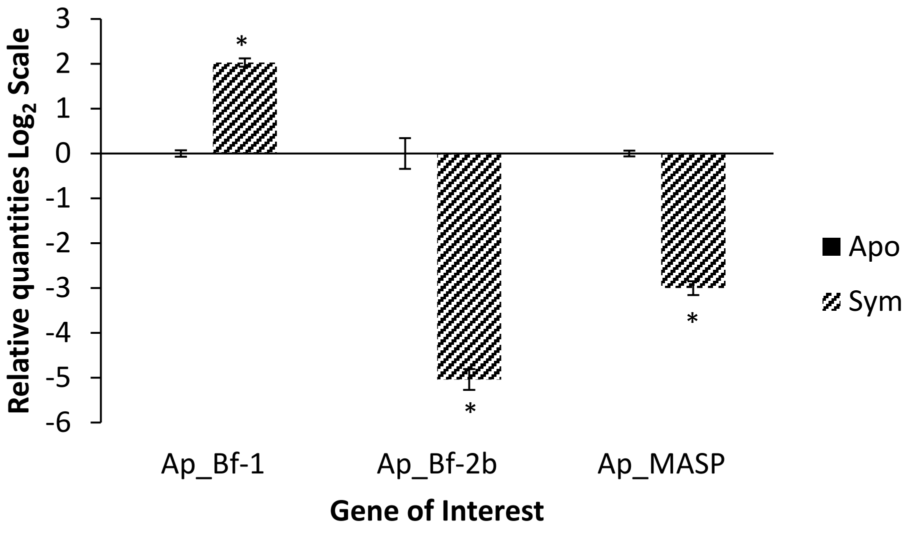

Supplementary Figure 1. Expression of Ap_Bf-1, Ap_Bf-2b, and Ap_MASP as a function of symbiotic state at time zero for the *S. marcescens* challenge. The relative quantities from qPCR on the $\log_2$ scale are shown for aposymbiotic organisms (solid bars) and symbiotic organisms (bars with lines). Bars represent means ±SE (n=3). Stars represent significant differences in expression across symbiotic states (1-tailed Wilcoxon rank-sum test * indicates p = 0.05)
